# Synthesis and anti-prion aggregation activity of acylthiosemicarbazide analogues

**DOI:** 10.1080/14756366.2023.2191164

**Published:** 2023-03-23

**Authors:** Dong Hwan Kim, Jaehyeon Kim, Hakmin Lee, Dongyun Lee, So Myoung Im, Ye Eun Kim, Miryeong Yoo, Yong-Pil Cheon, Jason C. Bartz, Young-Jin Son, Eun-Kyoung Choi, Yong-Sun Kim, Jae-Ho Jeon, Hyo Shin Kim, Sungeun Lee, Chongsuk Ryou, Tae-gyu Nam

**Affiliations:** a Department of Pharmacy and Institute of Pharmaceutical Science and Technology, Hanyang University ERICA campus, Ansan, Republic of Korea; b Division of Developmental Biology and Physiology, Department of Biotechnology, Sungshin University, Seoul, Korea; c Department of Medical Microbiology and Immunology, School of Medicine, Creighton University, Omaha, NE, USA; d Department of Pharmacy, Sunchon National University, Suncheon, Republic of Korea; e Ilsong Institute of Life Science, Hallym University, Seoul, Republic of Korea; f Department of Biomedical Gerontology, Graduate School of Hallym University, Chuncheon, Korea

**Keywords:** Prion disease Creutzfeldt–Jakob disease, prion aggregation formation assay, acylthiosemicarbazide

## Abstract

Prions are infectious protein particles known to cause prion diseases. The biochemical entity of the pathogen is the misfolded prion protein (PrP^Sc^) that forms insoluble amyloids to impair brain function. PrP^Sc^ interacts with the non-pathogenic, cellular prion protein (PrP^C^) and facilitates conversion into a nascent misfolded isoform. Several small molecules have been reported to inhibit the aggregation of PrP^Sc^ but no pharmacological intervention was well established thus far. We, here, report that acylthiosemicarbazides inhibit the prion aggregation. Compounds **7x** and **7y** showed almost perfect inhibition (EC_50_ = 5 µM) in prion aggregation formation assay. The activity was further confirmed by atomic force microscopy, semi-denaturing detergent agarose gel electrophoresis and real-time quaking induced conversion assay (EC_50_ = 0.9 and 2.8 µM, respectively). These compounds also disaggregated pre-existing aggregates *in vitro* and one of them decreased the level of PrP^Sc^ in cultured cells with permanent prion infection, suggesting their potential as a treatment platform. In conclusion, hydroxy-2-naphthoylthiosemicarbazides can be an excellent scaffold for the discovery of anti-prion therapeutics.

## Introduction

Prions are the infectious protein that cause prion diseases, including bovine spongiform encephalopathy, scrapie, and Creutzfeldt–Jakob disease (CJD)^1^^,^^2^. The clinical signs of prion diseases are related to impaired brain function, such as cognitive dysfunction, cerebral ataxia and motor dysfunction^1^^,^^3^. The neuropathological features of prion diseases include spongiform degeneration and gliosis in accociation with the accumulation of PrP^sc^ in the brain^4^. Recent studies at the cellular and molecular levels report that spongiosis and neurodegeneration are caused by prion-induced chronic endoplasmic reticulum (ER) stress leading to the depletion of an intracellular lipid molecule and impaired lysosomal trafficking in brain cells^5^^,^^6^.

The ultimate pathogens, prions, are believed to be an aggregate of disease-causing prion protein (PrP^Sc^), which is the misfolded isoform of normal cellular prion protein (PrP^C^). In the propagation mechanism of prion diseases, PrP^Sc^ induces the conformational change of normal PrP^C^ to become a nascent misfolded PrP^Sc^ molecule^7^^,^^8^, demonstrating its infectious nature. Therefore, PrP^Sc^ acts as a catalyst or template in this transformation to propagate the misfolded conformer. PrP^Sc^ is capable of forming aggregates that further afford amyloids or fibril structures^9^. Recent studies of PrP fibrils using cryo-electron microscopy demonstrated that PrP^Sc^ molecules adopt parallel in register intermolecular β-sheet amyloid structures^10^^,^^11^.

**Figure 1.**
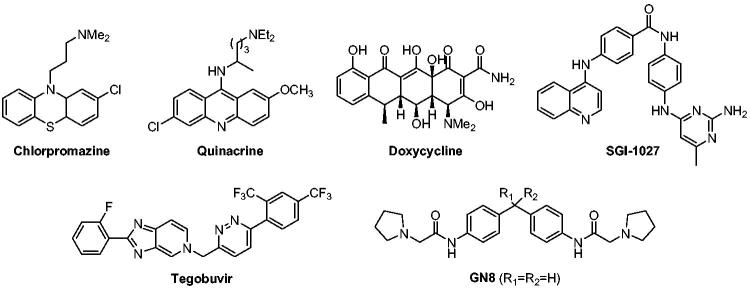
Representative compounds with anti-prion aggregation activity.

Since PrP conversion and aggregation occur autocatalytically without involvement of a signalling pathway or enzymatic system^9^^,^^12^, PrP^Sc^ molecule itself has been a direct drug target to interfere disease-causing aggregates^13–16^. Similarly, PrP^C^ also has been a target to block PrP conversion^17–25^. In addition, an auxiliary factor that assists in the PrP conversion was targeted to block the conversion^26–31^. Regardless of targets, several small molecules have been reported to inhibit PrP^Sc^ propagation (Figure 1)^32^. Some of them were identified through library screening, medicinal chemistry, or *in silico* approaches. For example, GN8 and its analogues (R_1_–R_2_=cycloheptyl) were discovered by a compound screening and subsequent medicinal chemistry efforts^21^^,^^22^. On the other hand, many compounds that provided intriguing anti-prion activity were known drugs or literature compounds that have irrelevant indications. They include chlorpromazine (antipsychotic)^23^, quinacrine (antimalarial)^33^, doxycycline (antibiotic)^15^, tegobuvir (antihepatitis C)^24^, and SGI-1027 (DNA methyltransferase inhibitor)^20^^,^^25^. None of them, however, have shown robust *in vivo* and clinical activity thus far, suggesting tremendous medical unmet needs for the discovery of new anti-prion agents.

In search of compounds to intervene misfolding pathologies, we have synthesised a series of acylthiosemicarbazide compounds some of which were found to inhibit ER stress as chemical chaperones^34^. ER stress is triggered by the abnormal accumulation of misfolded protein in ER. Inhibitors of ER stress relieve misfolded protein load either by decreasing the formation of misfolded proteins or by removing them by ER stress-associated degradation or apoptosis^35–38^. Because of the misfolding nature of PrP^Sc^, it would be worth investigating the cross-activity of the compounds. In this study, the scope of the analogues was expanded and their anti-prion activity of was measured by using prion aggregate formation assay (PAFA)^39^^,^^40^ that we have fine-tuned for the purpose of screening. Several compounds showed significant inhibition against prion aggregation. Some of them showed the activity to disassemble pre-existing aggregates. These activities were further confirmed by atomic force microscopy (AFM), semi-denaturing detergent agarose gel electrophoresis (SDD-AGE), and real-time quacking-induced conversion (RT-QuIC) assay with different prion strains. Also, the activity of compounds was evaluated in cultured cells with chronic prion infection.

## Materials and methods

### Synthesis of compounds

Synthetic details and spectroscopic data of new compounds are provided in the supplementary material.

### Preparation of recombinant PrP

The recPrP preparation was conducted according to the method described elsewhere^41^. Briefly, the cDNA fragments encoding His-tagged mouse or bank vole PrP (23–231) was cloned into plasmid pET100/D-TOPO (Invitrogen, Carlsbad, CA, USA). The recombinant plasmid was used for transformation of *E. coli* BL21 Star (DE3) (Invitrogen). Expression of recPrP was induced using 1 mM isopropyl β-D-1-thiogalactopyranoside (iNtRON, Seongnam-si, Gyeonggi-do, Korea). After incubation for 16 h, the bacterial cells were lysed by sonication in lysis buffer [10% sucrose (Sigma-Aldrich, St. Louis, MO, USA)], 0.1 M Tris (VWR Life Science, West Chester, PA, USA), 50 mM ethylenediaminetetraacetic acid (EDTA, pH 8.0) (Invitrogen), 0.2 M sodium chloride (NaCl) (pH 7.9) (Sigma-Aldrich), 1 mM phenylmethylsulfonylfluoride (MP Biomedicals, Santa Ana, CA, USA)]. The inclusion bodies derived from cell lysate were solubilised and refolded using solubilisation buffer [8 M urea (Samchun, Seoul, Korea), 10 mM glycine (Sigma-Aldrich), pH 10.6] and refolding buffer [0.6 M urea, 10 mM glycine, pH 10.6], respectively. The his-tagged mouse and bank vole recPrP was purified by sequential chromatography steps including immobilised metal affinity, cation exchange, and reverse phase chromatography. The purified mouse and bank vole recPrP was desalted and dissolved in 20 mM Tris, 150 mM NaCl, pH 8.0. Finally, recPrP was stored at −80 °C after adding glycerol (Sigma-Aldrich) and dimethyl sulfoxide (Sigma-Aldrich) 10% and 2%, respectively, to maintain stability.

### PrP aggregate formation assay

PAFA was conducted according to the method described elsewhere^39^. Ten µg mouse recPrP was denatured to unfolded using 6 M guanidine hydrochloride (GdnHCl) (Sigma-Aldrich) for 5 min at room temperature and then mixed in 200 µl reaction buffer [4M GdnHCl (Sigma-Aldrich) and 10 µM ThT (Sigma-Aldrich) in phosphate buffered saline (PBS, pH7.4) (Welgene, Gyeongsan-si, Gyeongsangbuk-do, Korea)] together with compounds (1–100 µM) and vehicle, DMSO. The PAFA reaction mixture was prepared in a 96-well black flat bottom polystyrene not treated microplate (Corning, Corning, NY, USA), sealed with a microplate adhesive film (USA Scientific, Ocala, FL, USA) and incubated at 37 °C for 60 h with shaking at 335 rpm in Infinite M200 Pro Fluorescence reader (Tecan, Maennedorf, Zurich, Switzerland). The fibrils formation was measured every 1 h by top reading of the fluorescence intensity using a 444 nm excitation and 485 nm emission filter.

### Atomic force microscopy

AFM was conducted according to the modified method described elsewhere^42^. Ten-mm-diameter highest grade V1 AFM mica discs (Ted Pella, Redding, CA, USA) was coated with 100 µl of 1% 3-aminopropyltriethoxysilane (Sigma-Aldrich) in 1 mM acetic acid (Sigma-Aldrich) for 20 min for fibrils absorption. After removing the solution, the mica was rinsed three times with 100 µl of distilled water, dried for 30 min using compressed N_2_ gas, and stored in a cryo box for 1 h. PAFA products were dropped onto the coated mica and incubated for 30 min until the fibrils were absorbed. After removing the solution, the mica was rinsed, dried, and stored in a cryo box as described above until imaging. Fibrils were imaged using XE-100 AFM (Park Systems, Suwon-si, Gyeonggi-do, Korea). To minimise damage to fibrils, imaging was performed in a non-contact mode using a point probe plus non-contact/tapping mode high resonance frequency reflex coating (PPP-NCHR) (Park Systems). During measurement, the constant scan rate was maintained at 0.5 Hz. The scan size was 10 µm × 10 µm to 1.5 µm × 1.5 µm, and the pixel was 256 × 256. AFM scanning was performed using XEP software (Park Systems), and the scanned image was edited using XEI software (Park Systems).

### Semi-denaturing detergent-agarose gel electrophoresis and western blotting

SDD-AGE was conducted according to the method described elsewhere with minor modification^43^. The medium for SDD-AGE was prepared with 1.5% agarose (Lonza, Basel, Switzerland) and 0.1% sodium dodecyl sulphate (SDS) (Sigma-Aldrich) in TAE (1 M Tris, 5.71% acetic acid, 50 mM EDTA) buffer. The horizontal gel was casted using an electrophoresis tray (Fisher scientific, Waltham, MA, USA). For sample preparation, 100 µl of PAFA products was centrifuged at 43 000 rpm for 1 at 4 °C h using ultracentrifuge TLX-100 (Beckman, Brea, CA, USA). The pellet was washed with 700 µl of PBS and retrieved by centrifugation at 43 000 rpm for 10 min at 4 °C. Then, the pellet was dissolved in 24 µl of SDD buffer [100 mM Tris (pH 7.5), 50 mM NaCl, 10 mM β-mercaptoethanol (Sigma-Aldrich)] and mixed with 8 µl of 4× sample loading buffer [2× TAE, 20% glycerol, 8% SDS, 0.3 M bromophenol blue (Sigma-Aldrich)]. After incubation at 37 °C for 10 min, the samples were run for 3 h at 60 V. Capillary transfer was conducted overnight using TBS [140 mM NaCl, 2 mM KCl (Sigma-Aldrich), 25 mM Tris, pH 7.4] onto a methanol (Honeywell)-activated 0.45 µm polyvinylidene difluoride (PVDF) (Merck, Piscataway, NJ, USA) membrane using the sequential piles of a sheet of TBS-wet extra thick blot filter paper (Bio-Rad, Hercules, CA, USA), 20 sheets of cellulose chromatography paper (Cytiva, Emeryville, CA, USA), and a 1 kg weight. After transfer, PVDF were subsequently blocked using 5% skim milk (BD Difco, Franklin Lakes, NJ, USA) in TBST [TBS, 0.5% Tween 20 (Sigma-Aldrich)] for 1 h. After rinsing in TBST, the membranes were incubated with anti-PrP monoclonal antibody 6D11 (Biolegend, San Diego, CA, USA) diluted to 1:30 000 in TBST for 1 h before washing in TBST for 10 min twice. Then, the membrane was further incubated with the secondary antibody (goat anti-mouse IgG HRP, Invitrogen) diluted 1:10 000 in TBST for 1 h. Following washing twice in TBST for 10 min, the membranes were developed using Amersham ECL prime (Cytiva) and visualised using a G:BOX Chemi XR5 Chemiluminescencer (Syngene, Cambrdige, UK).

### Disassembling assay for pre-formed PrP aggregates

Generation of pre-formed PrP aggregates was achieved by PAFA using mouse recPrP. When the formation of PrP aggregation was reached to the equilibrium after ∼32 h-PAFA reactions, the compounds with various concentrations in 10 µl were injected to each well, and the changes of aggregate-bound ThT fluorescence were further monitored for additional 20 h in the Infinite M200 Pro Fluorescence reader (Tecan, Maennedorf, Zurich, Switzerland) as described in the PAFA section.

### Real-time quaking-induced conversion

RT-QuIC was conducted according to the method described elsewhere with modifications^44^^,^^45^. Ten µg mouse or bank vole recPrP, prion seeds (5 × 10^−6^ diluted prion-ill rodent or human patient brain material), and compounds for anti-prion activity tests were mixed in 100 µl of RT-QuIC buffer [0.5M NaCl, 10 µM EDTA, 10 mM phosphate buffer (pH 7.4) (Sigma-Aldrich), 10 µM ThT, 0.002% SDS]. The source of prion seeds was FVB mouse-adapted RML scrapie prions, Syrian golden hamster-adapted Hyper TME prions and sporadic CJD prions deposited in Korea CJD Diagnostic Centre (Seoul, Korea). Prion seeds were prepared from 10% (w/v) homogenate of perfused brains by serial dilution with PBS. The reaction mixture in a 96-well Black/Clear Flat Bottom Polystyrene Not Treated Microplate (Corning) sealed with a microplate adhesive film (USA Scientific) was incubated at 42 °C for 60–80 h with shaking at 700 rpm in FLUOstar Omega Fluorescence reader (BMG Labtech, Ortenberg, Germany). Fibrils formation was measured every 1 h by bottom reading of ThT fluorescence intensity using a 450 nm excitation and 480 nm emission filter.

### Cytotoxicity and PrP^Sc^ assay in ScN2a cells

The cytotoxicity tests were conducted as described elsewhere using the MTT assay^46^. Briefly, ScN2a cells were incubated with the compounds for 4 days and the level of formazan formation facilitated by cellular enzymes were measured using MTT agents. PrP^Sc^ assay was performed in ScN2a cells as described elsewhere^47^. Briefly, ScN2a cells were incubated with the non-cytotoxic concentrations of compounds for 4 days and the proteinase K (PK)-resistant PrP^Sc^ in the cell lysate was detected by western blotting using anti-PrP monoclonal antibody 6D11 (Biolegend). Densitometry of western blots was performed using GeneTools software (Syngene). Student t-test was used for statistical analysis.

## Results and discussion

### Chemistry

**Scheme 1.**
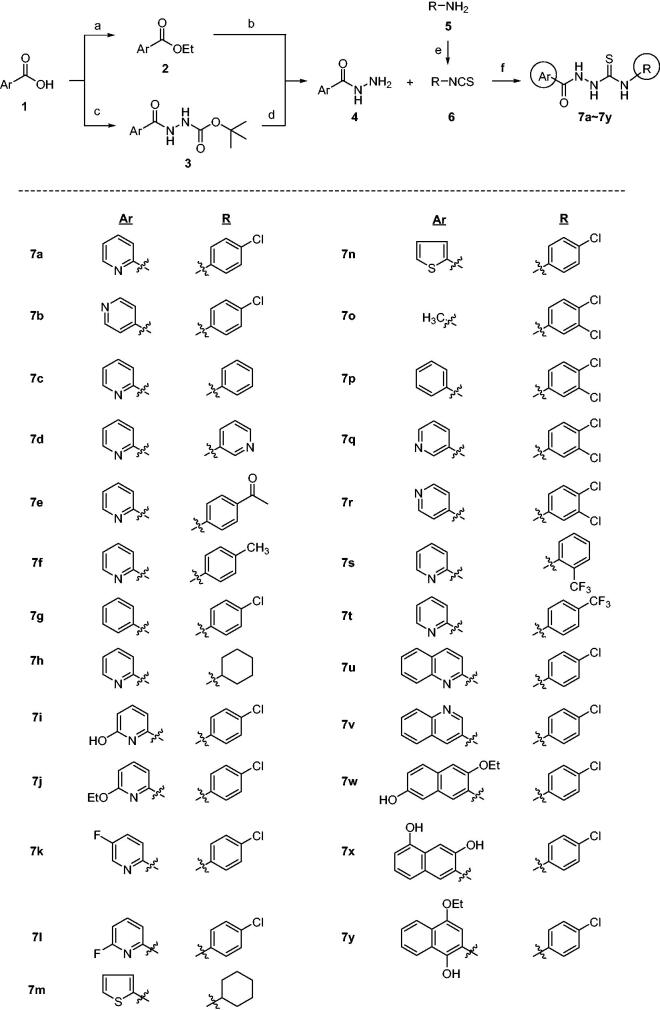
Synthesis of **7a–7y** with various Ar and R groups. *Reagents and conditions*: (A) H_2_SO_4_, EtOH, reflux, (B) H_2_NNH_2_H_2_O, EtOH, 80 °C, (C) H_2_N-NHt-Boc, HOAt, EDCl, NaHCO_3_, DMF, (D) 4 N HCl, dioxane, (E) CSCl_2_, Et_3_N, THF, 0 °C, (F) EtOH, r.t.

The synthesis of compounds **7** is shown in Scheme 1^34^. Acylhydrazides **4** were prepared from acid **1** via the reaction sequence consisting of either Fischer esterification followed by hydrazine substitution or coupling with t-butyl carbazate followed by hydrolysis of t-Boc group of compound **3**. Then, compounds **4** were reacted with isothiocyantes **6** to afford acylthiosemicarbazides **7** in 37–96% yields. When **6** is not commercially available, amines **5** were reacted with thiophosgene to give corresponding isothiocyanates **6**. Ar group includes phenyl, thienyl, (substituted)pyridyl, quinolinyl and (substituted)naphthyl while R group covers cycloalkyl, (substituted)phenyl, and (substituted)pyridyl groups.

**Scheme 2.**
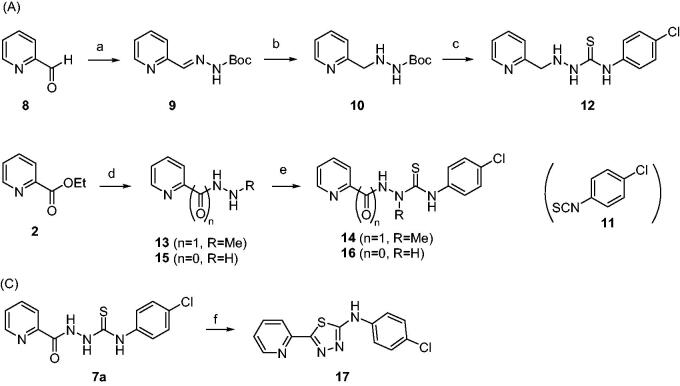
Synthesis of analogues with modifications in thiosemicarbazide moiety of **7a.** *Reagents and conditions*: (A) NH_2_NHBoc, Et_2_O, r.t., 1 day, 70%, (B) H_2_, Pd/C, MeOH, (C) (i) 4 N HCl-dioxane, 0 °C, 4 h, (ii) **11**, Et_3_N, EtOH, r.t., 4 h, 20%, (D) NH_2_NHCH_3_⋅H_2_SO_4_, Et_3_N, EtOH, reflux, 5 days, 46%, (E) **11**, EtOH, r.t., 4 h, 36% (for compound **14**), 77% (for compound **16**), (F) H_2_SO_4_ or HCl, EtOH, 0 °C → r.t., 2 h, 41%.

Syntheses of **12**, **14**, and **16** are depicted in Scheme 2. Unlike compounds **7a–7y** in which Ar and R groups are modified, these compounds have modifications in thiosemicarbazide moiety of **7a** that showed the highest level of anti-ER stress activity^34^. Structure–activity relationship (SAR) of **7a** could be elucidated by these analogues. Picolinaldehyde **8** was reacted with t-butyl carbazate to give compound **9** in 70% yield whose imine functional group was reduced to afford **10**. Deprotection of t-Boc group of **10** followed by a reaction with **11** yielded compound **12**, a reduced carbonyl version of **7a** (Scheme 2(A)). Meanwhile, reaction of ethyl ester **2** with hydrazine gave **13** which was then coupled with **11** to give **14**, an *N*-methyl analogue of **7a.** A coupling reaction of **15** with **11** afforded compound **16**, a decarbonyl version of **7a** (Scheme 2(B)). Also, compound **17**, a cyclic form of **7a**, was also prepared under acidic condition to investigate the effect of structural rigidity on the activity (Scheme 2(C)).

### Anti-prion aggregation activity

**Figure 2.**
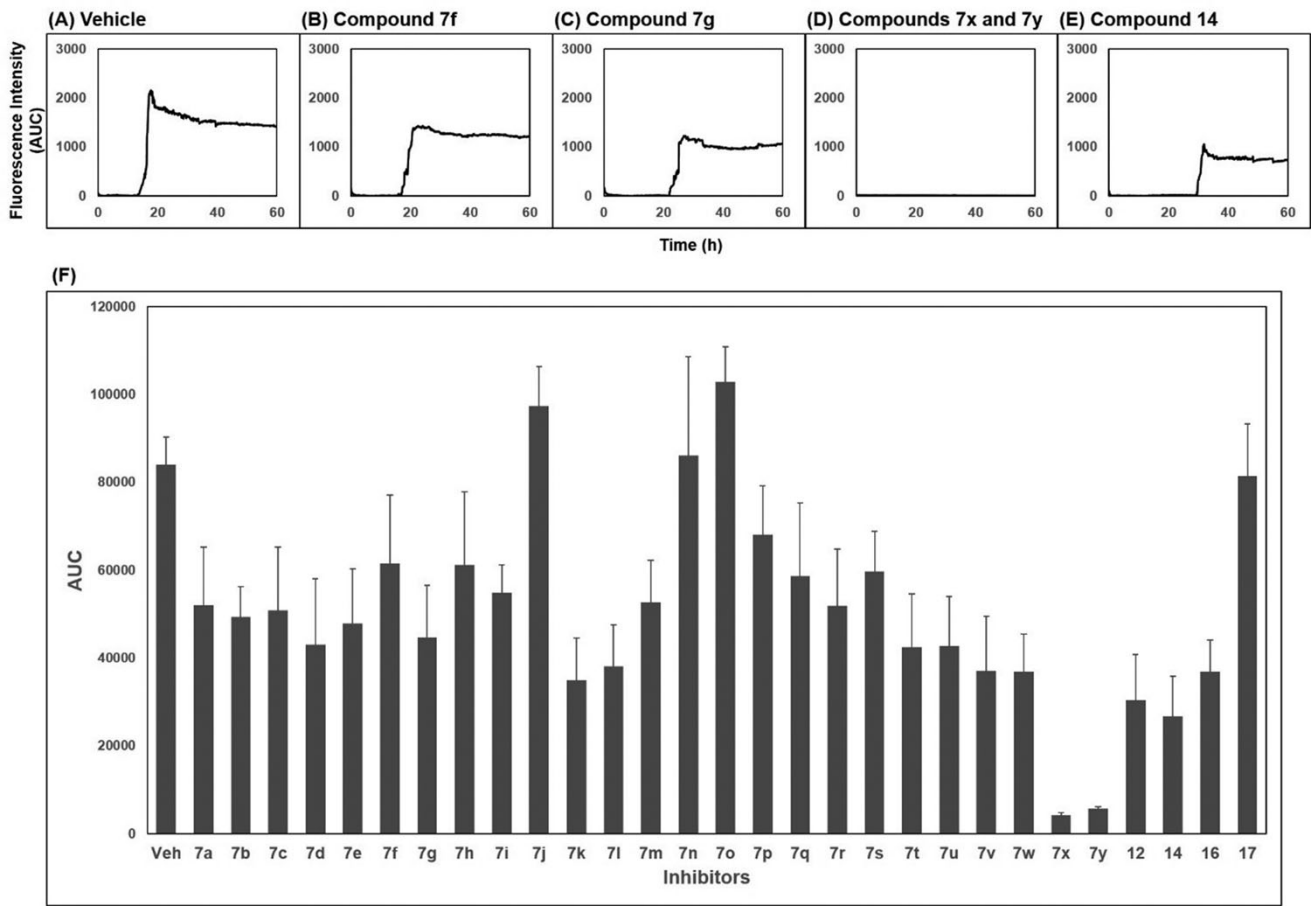
Area under the curve (AUC) of fluorescence curves obtained from PAFA. (A) vehicle condition. (B–E) Representative fluorescence curves by **7f**, **7g**, **7x**, **7y**, and **14**, respectively. (F) Anti-prion activity of the compounds measured by PAFA. AUC is expressed as mean ± SEM of independent experiments (*n* = 9). Veh = vehicle condition where DMSO was treated instead of 100 µM compounds.

The activity of compounds **7a–7 y**, **12**, **14**, **16**, and **17** inhibiting the formation of unfolded PrP aggregates was evaluated by PAFA. This assay monitors the aggregation of unfolded PrP based on the fluorescence emitted from aggregate-bound thioflavin T (ThT)^39^^,^^40^^,^^48^. Recombinant (rec) PrP, an initial component in a test tube, undergoes conformational change induced by guanidine HCl, a protein denaturant, into a partially molten form of PrP. This unfolded PrP conformer is subject to amyloid formation through self-aggregation and ThT binds to the resultant amyloids (Figure 2(A), vehicle condition). In PAFA, the anti-prion aggregation activity of compounds can be expressed in different aspects of fluorescence curves^49^. As the aggregation process requires a lag period to onset, some inhibitors can prolong the lag time and reduce the degree of aggregation (Figure 2(B,C,E)). Next, although the fluorescence level increases once the aggregation initiates at any delayed time points, other inhibitors can modulate the kinetics of aggregation or the level of plateau as each compound inhibits the aggregation at different efficacies (Figure 2(C,E)). When significantly active, the inhibitors can show marginal increase in fluorescence resulting in an almost flat curve (Figure 2(D)). Taken these aspects into account, area under the curve (AUC) of fluorescence curves can be a suitable readout of PAFA to represent the overall efficiency of the inhibitor.

Recombinant PrP used in this study contains His tag and theoretically the activity of a certain protein should be determined in the absence of any tags that are unnatural. A study reported that the presence of His tag caused technical difficulties in protein purification due to aggregation^50^. Hence, it was followed by some RT-QuIC studies that used recPrP without the tag. However, it is rather controversial as far as we are concerned. Our in-house experiences demonstrated the presence of His tag did not seriously affect the aggregate formation of recPrP^39^^,^^49^. This was confirmed by an independent study in which PrP aggregation reactions were conducted without the removal of the tag from PrP^51^. Moreover, Yen et al.^52^ showed the presence of the tag did not affect the secondary structure of PrP and PrP aggregation. At any rate, the activity of compounds that we report here is not questionable because the experiments were conducted under the identical condition using recPrP with the tag in all reactions including the vehicle control that showed a decent aggregation pattern. Then, the next concern is that whether the His tag could affect the interaction of the compounds with recPrP. Usually, the His tag shows a strong binding with divalent ions such as Ni^2+^ and similar sorts. Chemical structures of the active compounds clearly demonstrated that they do not contain such moieties. Thus, we can put off the verification of this issue and go forward to proving the anti-prion activity of compounds in other assays in cultured cells

**Figure 3.**
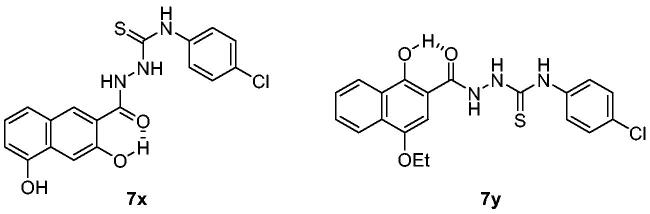
Potential cyclic structures through an internal hydrogen bond in **7x** and **7y**.

The activity of the compounds displayed in Figure 2 shows that most compounds have weak to moderate activity against aggregation. A few analogues of **7** and **7a** (**7k**, **7l**, **7v**, **7w** and **12**, **14**, **16**, respectively) have slightly better activity. Two compounds, **7x** and **7y**, showed particularly strong and robust anti-aggregation activity. SAR revealed that analogues with monocyclic Ar showed low activity except that fluoropyridinyl analogues, **7k** and **7l**, were marginally more active. Analogues of **7a** (**12**, **14**, and **16**) showed better activity than the parent compound **7a**. These observations indicated that the modification of Ar or thiosemicarbazide moiety can be beneficial to the activity at least to some degree when Ar group is monocyclic. The analogues with bicyclic Ar groups showed an interesting SAR. Whereas **7 u, 7v** (Ar = quinoline), and **7w** (Ar = hydroxynaphthalene) showed similar activity to **7k** and **7l**, **7x**, and **7y** afforded AUC of 4176 ± 565 and 5624 ± 497, respectively, that are about 15 ∼ 20-fold better activity than the vehicle condition (83 940 ± 6304). The hydroxy group on naphthalene ring of **7x** and **7y** can be attributed to the high activity by providing a six-membered cyclic structure with the carbonyl group via an internal hydrogen bond (Figure 3). This speculation can be supported by the low activity of **7v** and **7w** in which such a cyclic conformation is hardly formed. Moreover, the low activity of **7w** strongly demonstrated the crucial role of regiochemistry of the hydroxy group. It was also suggested that R group has very limited effect on the activity. Taken together, **7x** and **7y** were chosen for further study.

**Figure 4.**
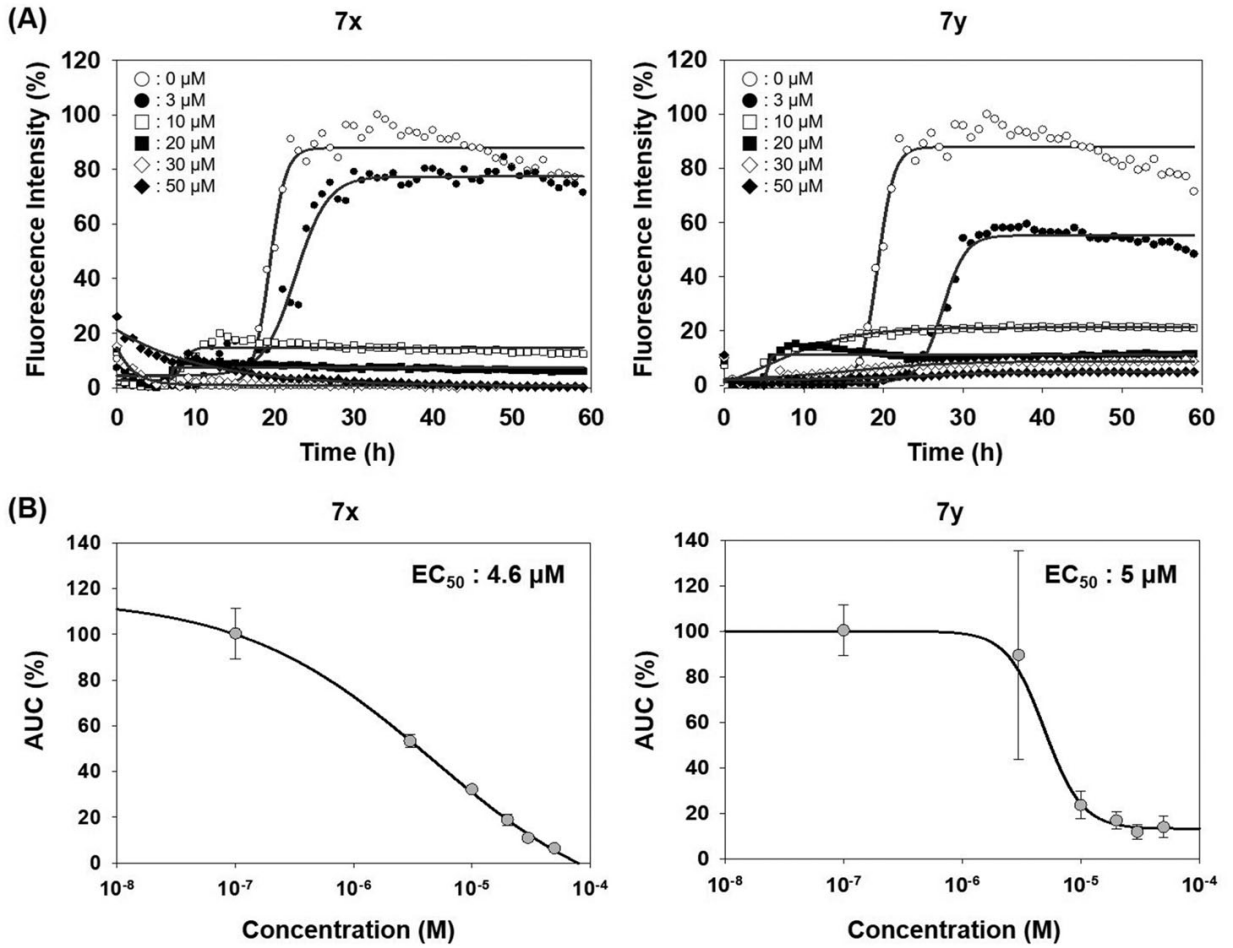
Activity of compounds to inhibit PAFA and dose–response curves. (A) PAFA with different concentrations of the compounds. The multiple PAFA reactions (*n* = 3) were performed and the mean of each data point was plotted. (B) Dose–response curves of the compounds based on mean AUC of each concentration from independent experiments (*n* = 3).

Compounds in this study were inspired by ER stress inhibitor scaffold that we have reported earlier^34^. ER stress is triggered when abnormal level of misfolded protein is accumulated in ER, which is responsible for the correct folding process during *de novo* protein synthesis. Activity of the compounds (EC_50_) against tunicamycin-induced ER stress was measured by a GRP78-driven reporter assay system^34^^,^^53^ and displayed in parallel with anti-prion aggregation activity (AUC) (Supplementary Figure 1). in order to investigate the correlation between two pathologies. Compounds **7a**, **7c–7f**, **7h**, **7k**, **7s**, and **7t** showed very strong inhibition against ER stress. SAR revealed that they all have 2-pyridyl moiety. It is speculated that pyridine N possibly form a cyclic conformation with the amide group via an internal hydrogen bond. Large differences in the inhibition between **7u** vs. **7v** and **7j** vs. **7i** can be explained in this context. Weak inhibition by **12**, **14**, **16**, and **17** compared to the parent compound **7a** demonstrated the essential role of the carbonyl group and NH of thiourea moiety in ER stress inhibition. Interestingly, in the case of the naphthalene Ar group, **7w** that cannot form the cyclic structure showed better ER stress inhibition than **7x** and **7y** that possibly form such conformation. The strong ER stress inhibitors, however, were not among the top-tier inhibitors in PAFA assay. Although both protein aggregation and ER stress are commonly linked with protein misfolding, compounds in this study showed a considerable discrepancy between the two activities. For example, **7x** and **7y,** the best anti-prion compounds, only showed marginal activity against ER stress (Supplementary Figure 1). Next, the dose-responsiveness of the anti-PrP aggregation activity of **7x** and **7y** was evaluated (Figure 4). Indeed, both compounds suppressed PrP aggregation in a dose-dependent manner. The dose–response curves based on the AUC at given concentrations determined EC_50_ = 4.6 µM and 5 µM for **7x** and **7y**, respectively.

**Figure 5.**
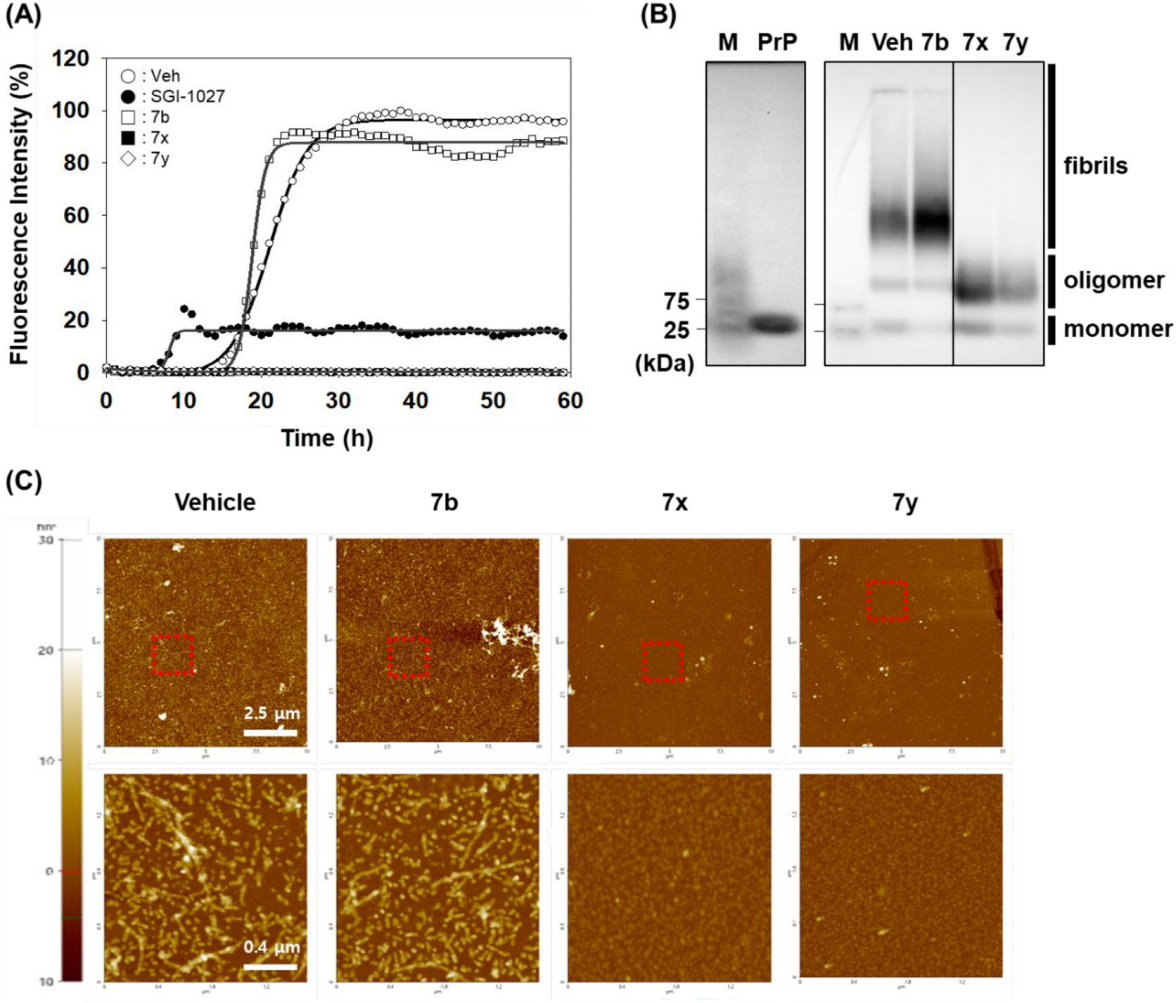
Biophysical states of PrP aggregates from PAFA. (A) PAFA with vehicle, positive control (SGI-1027), negative control (**7b**), and active compounds (**7x** and **7y**) at 10 µM each. The multiple PAFA reactions (*n* = 3) were performed and the mean of each data point was plotted. (B) SDD-AGE analysis of the reaction products of PAFA shown in panel A. M, molecular weight size marker; PrP, recPrP monomer form; Veh, vehicle. (C) Micrographs of the reaction products of PAFA shown in panel A by AFM. Dotted rectangles were magnified and displayed below.

The anti-PrP aggregation activity of **7x** and **7y** was further compared to that of SGI-1027 (a positive control). As shown in Figure 5(A), **7x** and **7y** significantly inhibited PrP aggregation in PAFA. The inhibitory activity was even better than that of the positive control, indicating the superiority of **7x** and **7y** as a PrP aggregation inhibitor, while **7b** (a negative control) remained almost inactive. The PAFA products of **7x** and **7y** were further examined for the biophysical state of PrP molecules using SDD-AGE and AFM. In SDD-AGE, the migration of most PrP aggregates from PAFA with vehicle and negative control **7b** was retarded, showing the massive band of very high molecular weights (Figure 5(B)). In contrast, the reaction products of PAFA with **7x** and **7y** showed a completely different band pattern of PrP molecules. Only PrP oligomers and monomers were observed, lacking PrP aggregates of very high molecular weights. In addition, as clearly shown in the AFM images of PAFA products, **7x** and **7y** significantly reduced the formation of fibrillar structure, while vehicle and **7b** mostly showed fibrils (Figure 5(C)). The examination of biophysical states of PrP molecules in PAFA verified that **7x** and **7y** did actually inhibit aggregation to remove fibrillar aggregates observed in vehicle condition. Moreover, these results indicate that fluorescence-based detection of PrP aggregation controlled by compounds in PAFA is a reliable and robust assay to measure the efficacy of an anti-PrP aggregation inhibitor. Nevertheless, it is cautious to conclude that **7x** and **7y** would be beneficial to modify the course of disease *in vivo* because the compounds were not able to completely degrade PrP aggregates of very high molecular weights into monomers, but rather induced the emergence of a considerable level of the oligomers, which could form PrP fibrils again and, more critically, could be more harmful to neurons than PrP fibrils and monomers^54^.

**Figure 6.**
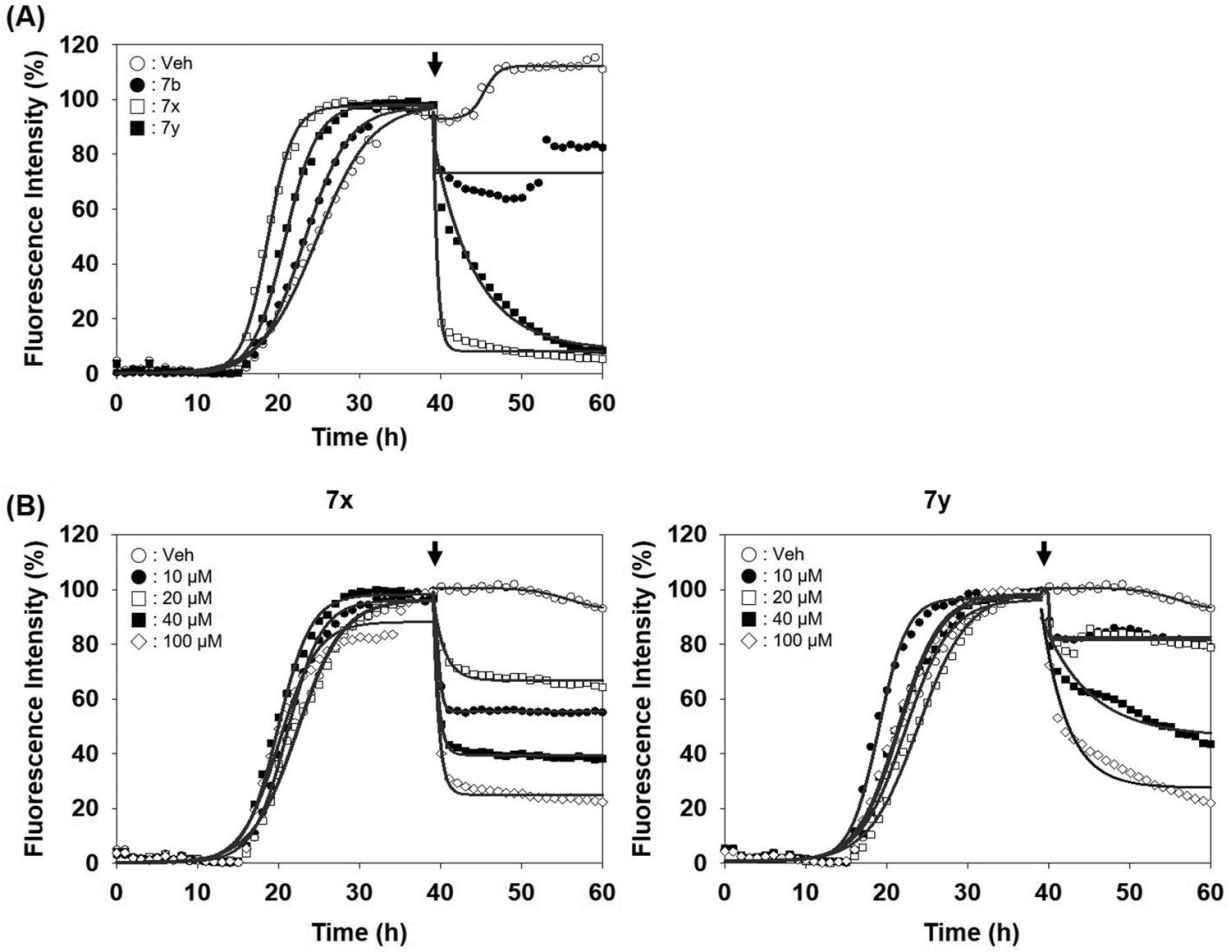
Activity of compounds to disassemble pre-existing aggregates. (A) Disaggregation curves of vehicle, negative control (**7b**), **7x**, and **7y** at 100 µM concentration. (B) Dose-dependent curves of **7x** and **7y** at different concentrations. Arrows indicate the time point of compound treatment. The multiple disaggregation reactions of PAFA products (*n* = 3) were conducted and the mean of each data point was plotted.

After identifying the inhibitors by PAFA that can measure the activity in a context of disease prevention, we sought to address whether they can exert the activity in a disease treatment setting as well. So, the compounds were tested if they disassemble pre-formed aggregates to smaller size aggregates or monomers. Each compound was spiked in the reaction after PrP aggregation reached the plateau in PAFA and the changes of PrP aggregation were monitored. As shown in Figure 6(A), the fluorescence level immediately decreased upon the injection of **7x** and **7y** after the completion of pre-formation of PrP aggregates. It indicates that they have significant disaggregation activity against the pre-formed aggregates, while compound **7b** showed no such effect. Also, the disassembling activity of the compounds was clearly confirmed in a dose-dependent manner (Figure 6(B)).

**Figure 7.**
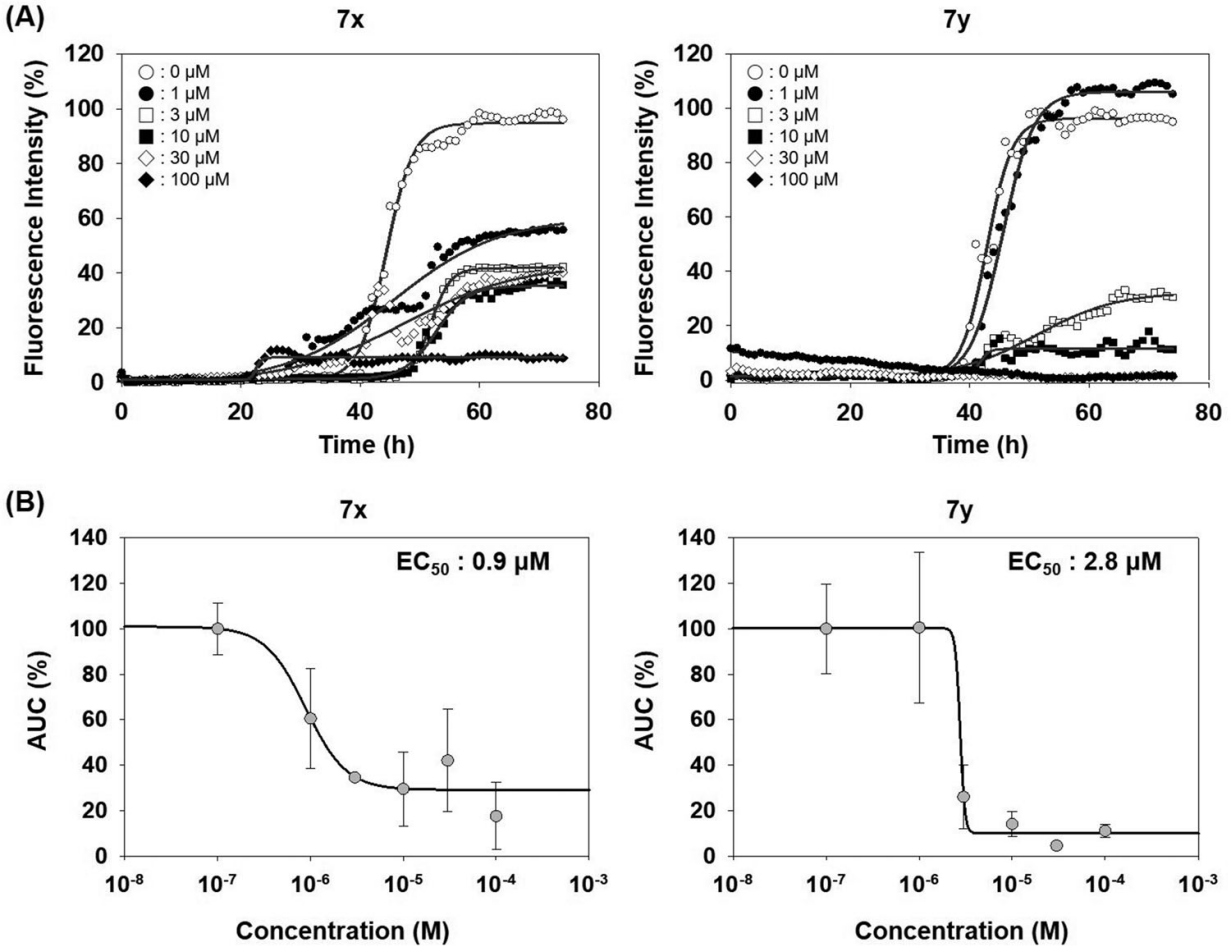
Activity of compounds to inhibit RT-QuIC and dose–response curves. (A) RT-QuIC assay at different concentrations of **7x** and **7y**. The assay was performed using mouse recPrP and 5x1 0 ^−6^ diluted RML-ill mouse brain homogenates. Each concentration line represents mean of multiple RT-QuIC reactions (*n* = 3). (B) Dose–response curves of the compounds based on AUC of each concentration from independent experiments (*n* = 2).

**Figure 8.**
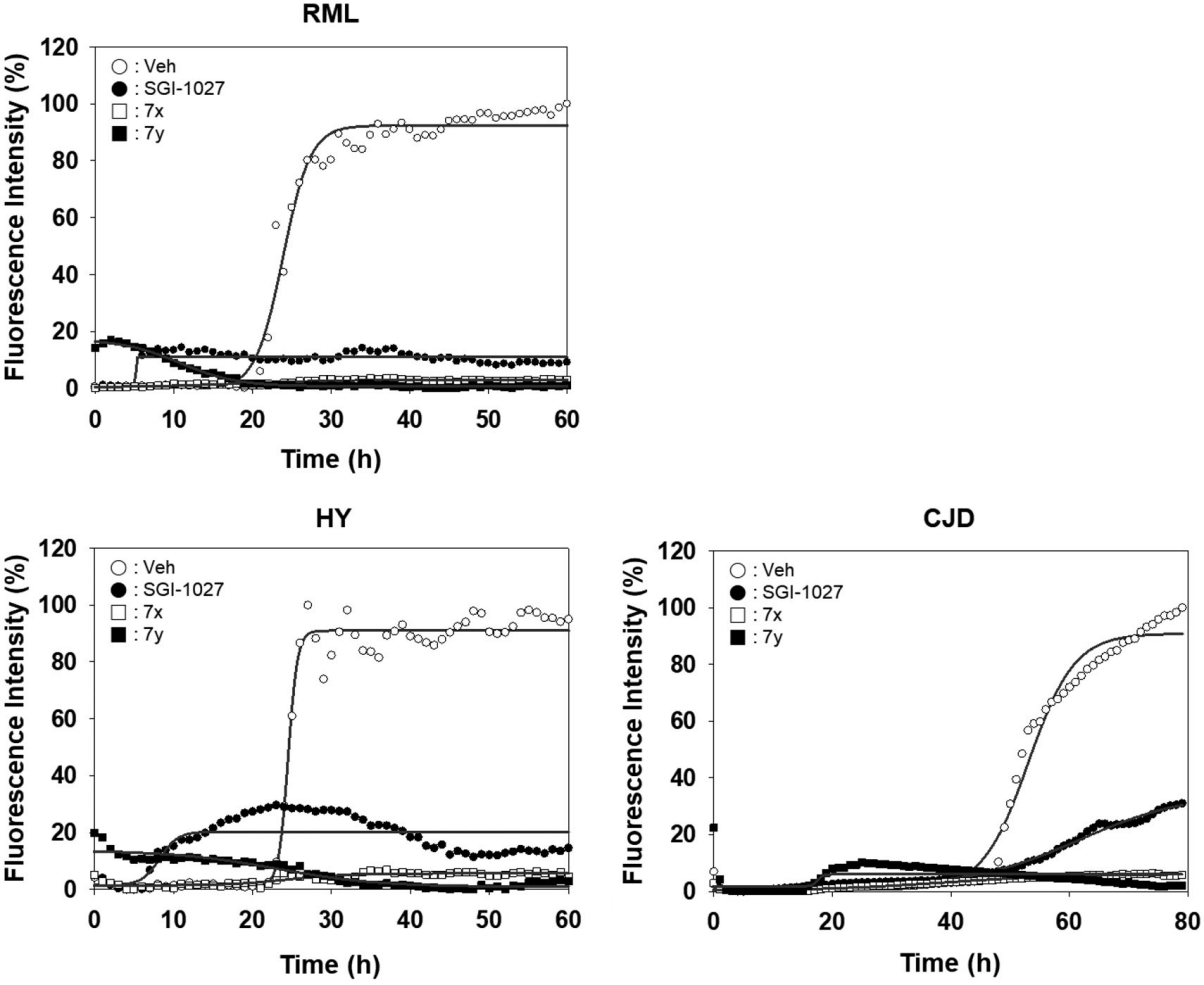
Activity of compounds to inhibit RT-QuIC performed with prions of various sources. Each RT-QuIC assay was performed using bank vole recPrP in the presence of 100 µM **7x** and **7y**. The prion seeds were 5 × 10^−6^ diluted brain homogenates of RML-ill mice, Hyper-ill Syrian hamsters and a CJD patient. Each concentration line represents mean of independent experiments (*n* = 3).

Unlike PAFA experiments in which PrP is partially unfolded by denaturants and self-aggregation of unfolded PrP occurs, the physiological observations indicate that PrP^C^ undergoes misfolding through conformational change by PrP^Sc^ and *de novo-*generated PrP^Sc^ becomes aggregated. The ability of compounds **7x** and **7y** to inhibit the PrP conversion facilitated by PrP^Sc^ seeding was measured using RT-QuIC assay (Figure 7)^44^^,^^45^^,^^49^. The formation of PrP aggregates by RML PrP^Sc^ seed-induced conversion of mouse recPrP to the misfolded conformer was inhibited by **7x** and **7y** in a concentration-dependent fashion (Figure 7(A)). AUC curves determined the inhibitory efficacy of **7x** and **7y** to be EC_50_ = 0.9 µM and 2.8 µM, respectively (Figure 7(B)). This result suggested that **7x** and **7y** were able to inhibit PrP^Sc^ aggregation preceded by PrP conversion. Next, the inhibitory ability of compounds **7x** and **7y** in RT-QuIC was investigated with prions of various sources. RT-QuIC using bank vole recPrP substrates and seeding with RML, Hyper, and CJD prions showed robust PrP conversion and following aggregation, and compounds **7x** and **7y** efficiently inhibited it (Figure 8). These results indicated that **7x** and **7y** are efficacious to a panel of prions from animals to humans.

**Figure 9.**
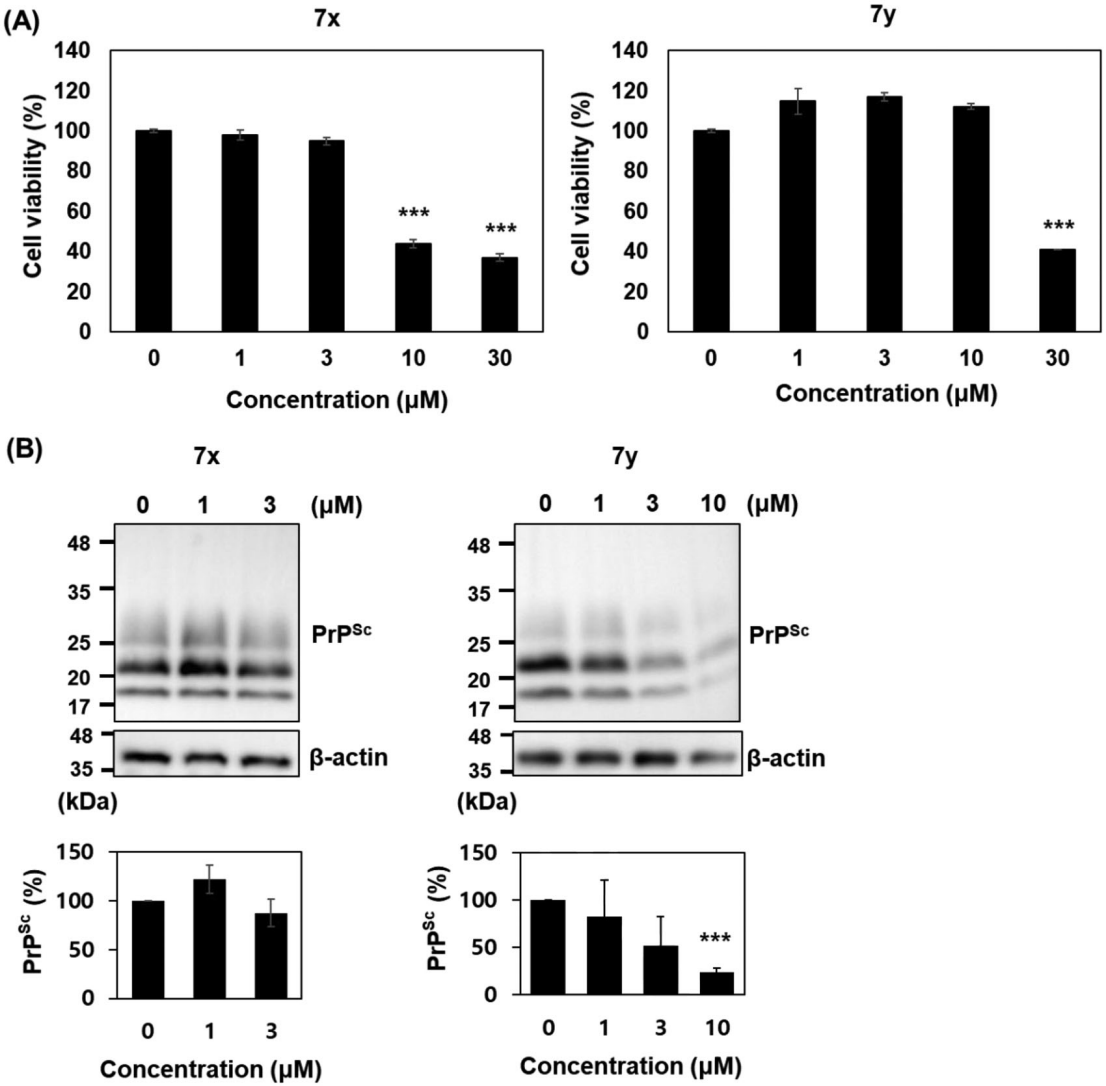
Cytotoxicity and anti-prion activity of compounds in ScN2a cells. (A) Cytotoxicity of **7x** and **7y**. The MTT assay were performed (*n* = 3) and the mean was plotted. (B) Western blots of PK-resistant PrP^Sc^ in the cells incubated with **7x** and **7y**. β-actin was used as a loading control. Western blotting was performed multiple times (*n* = 4). The PrP^Sc^ level of each blot was quantified by densitometry and the means were plotted. ***p < 0.01.

Anti-prion activity of **7x** and **7y** was further investigated in cultured cells with permanent prion infection. Cytotoxicity tests in ScN2a cells showed that **7x** was marginally more cytotoxic than **7y**: the survival of cells incubated with **7x** and **7y** was significantly reduced over 10 and 30 µM, respectively. The western blot detection of PK-resistant PrP^Sc^ level in ScN2a cells incubated with the non-cytotoxic concentrations of compounds showed that 10 µM **7y** decreased the PrP^Sc^ level over 75% compared to vehicle control, but **7x** failed (Figure 9).

In conclusion, here, we report the synthesis of acylthiosemicarbazide compounds and their activity to inhibit PrP conversion and aggregation. PAFA indicated that compounds with monocyclic Ar groups showed low-to-medium range activity against PrP aggregation. Compounds **7x** and **7y** that have hydroxy-2-naphthyl Ar group were the most active inhibitors to give EC_50_ ≅ 5 µM in PAFA that is 15 ∼ 20-fold better activity than vehicle. Their activity can be attributed to a cyclic conformation through the internal hydrogen bond. They also showed significant activity to disassemble pre-existing PrP aggregates. Their activities were further confirmed by AFM, SDD-AGE, and RT-QuIC assay, which is relevant to the physiological condition since PrP^Sc^ from infected animal and humans is involved and therefore mimics the essence of prion propagation. Both compounds showed even better efficacy, showing EC_50_ = 0.9 µM and 2.8 µM, respectively in RT-QuIC assay. Furthermore, one of compounds **7y** was effective to decrease the level of PrP^Sc^ in cultured cells with permanent prion infection. However, their anti-prion aggregation activity was not closely correlated with the anti-ER stress activity. Taken together, data suggested that SAR and inhibitors identified in this study can provide an excellent platform for the discovery of anti-prion therapeutics.

## Supplementary Material

Supplemental Material
