# Supplementary material for: Synthesis and anti-prion aggregation activity of acylthiosemicarbazide analogues
Source: J Enzyme Inhib Med Chem. 2023 Mar 23;38(1):2191164. doi: 10.1080/14756366.2023.2191164 (PMC10038035; doi:10.1080/14756366.2023.2191164)
Supplement: Supplemental Material [file IENZ_A_2191164_SM3466.pdf]

Synthesis and Anti-prion Aggregation Activity of Acylthiosemicarbazide Analogues

Dong Hwan Kim[1,†], Jaehyeon Kim[1,†], Hakmin Lee[1], Dongyun Lee[1], So Myoung Im[1], Ye Eun Kim[1], Miryoung Yoo[1], Yong-Pil Cheon[2], Jason C. Bartz[3], Young-Jin Son[4], Eun-Kyoung Choi[5], Yong-Sun Kim[5], Jae-Ho Jeon[1], Hyo Shin Kim[1], Seungeun Lee[1], Chongsuk Ryou[1,*] and Tae-gyu Nam[1,*]

[1]Department of Pharmacy and Institute of Pharmaceutical Science and Technology, Hanyang University, Ansan, Gyeonggi-do 15588, Republic of Korea

[2]Division of Developmental Biology and Physiology, School of Bioscience and Chemistry, Institute for Basic Sciences, Sungshin University, Seoul 02844, Republic of Korea

[3]Department of Medical Microbiology and Immunology, School of Medicine, Creighton University, Omaha, NE 68178, U. S. A.

[4]Department of Pharmacy, Sunchon National University, Suncheon 57922, Republic of Korea

[5]Ilsong Institute of Life Science, Hallym University, Seoul 07247, Republic of Korea

Correspondence:

Chongsuk Ryou: Tel.: +82 31 400 5811; Fax: +82 31 400 5958; E-mail: cryou2@hanyang.ac.kr

Tae-gyu Nam: Tel.: +82 31 400 5807; Fax: +82 31 400 5958; E-mail: tnam@hanyang.ac.kr

[†]These authors contributed equally to this work.

Contents

Synthetic details of new compounds -- 2

NMR spectrum of new compounds -- 9

Supplementary Figure 1 -- 31

General procedure for synthesis of compound 2

H_2SO_4 (1.2 mL) was added to a solution of acid **1** (20 mmol) in EtOH (150 mL). The mixture was refluxed overnight. The reaction mixture was concentrated, diluted with ethyl acetate, washed with $NaHCO_3$, water, brine, and dried over anhydrous $MgSO_4$. The residue was purified by using silica gel chromatography (1~10% EA in Hexanes) to give compound **2**.

Ethyl 3-ethoxy-7-hydroxy-2-naphthoate (2w)

Yield 59%. ^{1}H NMR (400 MHz, $CDCl_3$) δ 10.29 (s, 1H), 8.30 (s, 1H), 7.52 (d, J = 9.1 Hz, 1H), 7.12 (dd, J = 9.0, 2.5 Hz, 1H), 7.01 (d, J = 2.5 Hz, 1H), 4.41 (q, J = 7.1 Hz, 2H), 4.04 (q, J = 7.0 Hz, 2H), 1.43 – 1.38 (m, 6H). MS (ESI) *m/z*: 261 $[M+H]^+$.

General procedure for synthesis of compound 3

An acid **1** (1 mmol), tert-butyl hydrazinecarboxylate (159mg, 1.2 mmol) was dissolved in DMF (3 ml) at 0 °C. Then $NaHCO_3$ (210 mg, 2.5 mmol), HOAt (272 mg, 2 mmol), EDCI (310 mg, 2 mmol) was added. The mixture was stirred at r.t. for 1 day. The reaction mixture was diluted with EA, washed with 1 N HCl × 3 times, $NaHCO_3$ × 3 times, water, and brine, and dried over anhydrous $MgSO_4$. The organic layer was concentrated to give **3**.

tert-butyl 2-(5-fluoropicolinoyl)hydrazine-1-carboxylate (3k)

Yield 99%. ^{1}NMR (400 MHz, DMSO) δ 10.36 (s, 1H), 8.95 (s, 1H), 8.68 (d, J = 2.8 Hz, 1H), 8.12 (dd, J = 8.7, 4.6 Hz, 1H), 8.01 – 7.88 (m, J = 8.7, 2.8 Hz, 1H), 1.44 (s, 9H). MS (ESI) *m/z*: 256 $[M+H]^+$

tert-butyl 2-(6-fluoropicolinoyl)hydrazine-1-carboxylate (3l)

Yield 96%. ^{1}H NMR (400 MHz, DMSO) δ 10.37 (s, 1H), 8.97 (s, 1H), 8.22 (dd, J = 15.7, 8.1

Hz, 1H), 8.01 – 7.95 (m, 1H), 7.48 (dd, *J* = 8.2, 1.9 Hz, 1H), 1.44 (s, 9H). MS (ESI) *m/z*: 256 $[M+H]^+$

General procedure for synthesis of compound 4

(from compound 2) Hydrazine hydrate (0.5 mL, 16 mmol) was added to compound **2** (4 mmol) in EtOH (50 mL). The reaction mixture was stirred for 1 day at 80 °C. The mixture was concentrated, diluted with EA, washed with water, brine, and dried over anhydrous $MgSO_4$. The residue was purified by using silica gel chromatography to give compound **4**.

(from compound 3) A compound **3** (2 mmol) was dissolved in dioxane (8 mL) and 4 N HCl (8 mL). The reaction mixture was stirred at r.t. for 4 hours then was concentrated, diluted with EA, washed with water, brine, and dried over anhydrous $MgSO_4$. The residue was purified by using silica gel chromatography to give compound **4**.

6-hydroxypicolinohydrazide (4i)

Yield 83%. ^{1}H NMR (400 MHz, DMSO) δ 7.62 (dd, *J* = 8.7, 7.0 Hz, 1H), 7.05 (dd, *J* = 7.0, 0.9 Hz, 1H), 6.64 (dd, *J* = 8.7, 0.9 Hz, 1H). MS (ESI) *m/z*: 154 $[M+H]^+$

6-Ethoxypicolinohydrazide (4j)

Yield 83%. ^{1}H NMR (400 MHz, DMSO) δ 9.74 (s, 1H), 7.84 (dd, *J* = 8.3, 7.3 Hz, 1H), 7.56 (dd, *J* = 7.3, 0.8 Hz, 1H), 6.96 (dd, *J* = 8.3, 0.8 Hz, 1H), 4.57 (d, *J* = 4.3 Hz, 2H), 4.46 (q, *J* = 7.1 Hz, 2H), 1.33 (t, *J* = 7.1 Hz, 3H). MS (ESI) *m/z*: 182 $[M+H]^+$

3-Ethoxy-7-hydroxy-2-naphthohydrazide (4w)

Yield 90%. ^{1}H NMR (400 MHz, DMSO) δ 11.60 (s, 1H), 10.11 (s, 1H), 8.33 (s, 1H), 7.67 (d, *J* = 8.9 Hz, 1H), 7.30 – 7.08 (m, 3H), 4.74 (s, 2H), 4.12 (q, *J* = 7.0 Hz, 2H), 1.40 (t, *J* = 7.0 Hz,

3H). MS (ESI) *m/z*: 247 $[M+H]^+$

3,5-Dihydroxy-2-naphthohydrazide (4x)

Yield 86%. ^{1}H NMR (400 MHz, DMSO) *δ* 11.31 (s, 1H), 10.02 (s, 2H), 8.35 (s, 1H), 7.43 (s, 1H), 7.27 (d, *J* = 8.4 Hz, 1H), 7.12 (dd, *J* = 8.1, 7.6 Hz, 1H), 6.83 (dd, *J* = 7.4, 0.8 Hz, 1H), 4.73 (s, 2H). MS (ESI) *m/z*: 219 $[M+H]^+$

4-Ethoxy-1-hydroxy-2-naphthohydrazide (4y)

Yield 40%. ^{1}H NMR (400 MHz, DMSO) *δ* 13.95 (s, 1H), 10.28 (s, 1H), 8.29 – 8.21 (m, 1H), 8.12 (dd, *J* = 8.2, 0.7 Hz, 1H), 7.68 – 7.58 (m, 2H), 7.27 (s, 1H), 4.74 (s, 2H), 4.18 (q, *J* = 7.0 Hz, 2H), 1.47 (t, *J* = 7.0 Hz, 3H). MS (ESI) *m/z*: 247 $[M+H]^+$

General procedure for synthesis of compound 7

Isothiocyanates (**6**, 2.2 mmol) was added to a solution of **4** (2 mmol) in EtOH (20 mL). The mixture was stirred at r.t.~ reflux for 1 day. The reaction mixture was filtered and filter cake was washed with ether to give **7**.

2-Picolinoyl-N-(pyridin-3-yl)hydrazine-1-carbothioamide (7d)

Yield 56%. ^{1}H NMR (400 MHz, DMSO) δ 10.82 (s, 1H), 9.93 (d, *J* = 29.2 Hz, 1H), 9.85 (s, 1H), 8.69 (ddd, *J* = 4.8, 1.6, 1.0 Hz, 1H), 8.57 (s, 1H), 8.33 (dd, *J* = 4.7, 1.5 Hz, 1H), 8.10 – 8.06 (m, 1H), 8.03 (td, *J* = 7.6, 1.7 Hz, 1H), 7.89 (s, 1H), 7.68 – 7.63 (m, 1H), 7.36 (dd, *J* = 8.2, 4.7 Hz, 1H). ^{13}C NMR (100 MHz, DMSO) δ 181.7, 164.2, 149.8, 148.9, 147.3, 146.1, 138.2, 136.6, 133.6, 127.5, 123.3, 123.0. HRMS (ESI) m/z calculated for $C_{12}H_{12}N_5OS$ $[M+H]^+$ 274.0757, found 274.0763. MS (ESI) *m/z*: 274 $[M+H]^+$

N-(4-Acetylphenyl)-2-picolinoylhydrazine-1-carbothioamide (7e)

Yield 48%. ^{1}H NMR (400 MHz, DMSO) δ 10.81 (s, 1H), 9.96 (br s, 2H), 8.70 (d, *J* = 4.5 Hz, 1H), 8.10 – 8.06 (m, 1H), 8.03 (td, *J* = 7.6, 1.6 Hz, 1H), 7.91 (d, *J* = 8.6 Hz, 2H), 7.73 (d, *J* =

7.5 Hz, 2H), 7.66 (ddd, *J* = 7.2, 4.8, 1.5 Hz, 1H), 2.54 (s, 3H). ^{13}C NMR (100 MHz, DMSO) δ 197.3, 180.9, 164.2, 149.8, 149.0, 144.3, 138.2, 133.2, 128.8, 127.5, 124.8, 122.9, 27.0. HRMS (ESI) m/z calculated for $C_{15}H_{15}N_4O_2S$ $[M+H]^+$ 315.0910, found 315.0925. MS (ESI) *m/z*: 315 $[M+H]^+$

N-(4-Chlorophenyl)-2-(6-hydroxypicolinoyl)hydrazine-1-carbothioamide (7i)

Yield 42%. ^{1}H NMR (400 MHz, DMSO) δ 11.22 (s, 1H), 10.48 (s, 1H), 9.87 (s, 2H), 7.70 (s, 1H), 7.49 (d, *J* = 7.0 Hz, 2H), 7.43 – 7.34 (m, 2H), 7.19 (s, 1H), 6.76 (s, 1H). ^{13}C NMR (100 MHz, DMSO) δ 181.2, 162.7, 140.7 (2C), 138.6 (2C), 129.5, 128.5 (2C), 128.9, 128.8. HRMS (ESI) m/z calculated for $C_{13}H_{12}ClN_4O_2S$ $[M+H]^+$ 323.0286, found 323.0312. MS (ESI) *m/z*: 323 $[M+H]^+$

N-(4-Chlorophenyl)-2-(6-ethoxypicolinoyl)hydrazine-1-carbothioamide (7j)

Yield 87%. ^{1}H-NMR (400 MHz, DMSO) δ 10.60 (s, 1H), 9.84 (d, *J* = 34.1 Hz, 2H), 7.90 (dd, *J* = 8.2, 7.4 Hz, 1H), 7.65 (dd, *J* = 7.3, 0.8 Hz, 1H), 7.54 (d, *J* = 28.4 Hz, 2H), 7.39 (d, *J* = 8.8 Hz, 2H), 7.04 (d, *J* = 7.9 Hz, 1H), 4.52 (q, *J* = 7.0 Hz, 2H), 1.35 (t, „*J* = 7.1 Hz, 3H). ^{13}C-NMR (100 MHz, DMSO) δ 180.9, 166.4, 162.2, 146.8, 140.2, 138.3, 128.8, 127.8, 127.4, 115.6, 114.4, 56.0, 18.6. HRMS (ESI) m/z calculated for $C_{15}H_{16}ClN_4O_2S$ $[M+H]^+$ 351.0626, found 351.0659. MS (ESI) *m/z*: 351 $[M+H]^+$

N-(4-chlorophenyl)-2-(5-fluoropicolinoyl)hydrazine-1-carbothioamide (7k)

Yield 68%. ^{1}H-NMR (400 MHz, DMSO) δ 10.78 (s, 1H), 9.89 (s, 1H), 9.77 (s, 1H), 8.72 (d, *J* = 2.8 Hz, 1H), 8.17 (dd, *J* = 8.8, 4.6 Hz, 1H), 7.96 (td, *J* = 8.7, 2.9 Hz, 1H), 7.50 (s, 2H), 7.43 – 7.33 (m, 2H). ^{13}C-NMR (100 MHz, DMSO) δ 180.7, 162.1, 159.5, 146.1, 138.2, 136.9 (d, *J* = 20 Hz, 1C), 128.8, 127.8, 127.2, 124.8, 124.4 (d, *J* = 20 Hz, 1C). HRMS (ESI) m/z calculated for $C_{13}H_{11}ClFN_4OS$ $[M+H]^+$ 325.0269, found 325.0306. MS (ESI) *m/z*: 325 $[M+H]^+$

N-(4-chlorophenyl)-2-(6-fluoropicolinoyl)hydrazine-1-carbothioamide (7l)

Yield 35%. ^{1}H-NMR (400 MHz, DMSO) δ 10.77 (s, 1H), 9.83 (d, *J* = 39.9 Hz, 2H), 8.21 (d, *J* = 6.8 Hz, 1H), 8.01 (d, *J* = 5.9 Hz, 1H), 7.47 (d, *J* = 7.2 Hz, 3H), 7.37 (d, *J* = 8.2 Hz, 2H). ^{13}C-NMR (100 MHz, DMSO) δ 179.5, 161.8, 159.4, 146.9, 142.6, 127.8 (d, *J* = 43.5 Hz), 126.9, 123.0, 119.7, 117.8, 112.2 (d, *J* = 36.4 Hz). HRMS (ESI) m/z calculated for $C_{13}H_{11}ClFN_4OS$ $[M+H]^+$ 325.0269, found 325.0307. MS (ESI) *m/z*: 325 $[M+H]^+$

N-(3,4-dichlorophenyl)-2-isonicotinoylhydrazine-1-carbothioamide (7r)

Yield 57%. ^{1}H NMR (400 MHz, DMSO) δ 10.91 (s, 1H), 10.08 (s, 1H), 9.94 (s, 1H), 8.79 (dd, *J* = 4.4, 1.7 Hz, 2H), 7.85 (d, *J* = 6.0 Hz, 2H), 7.81 (s, 1H), 7.60 (d, *J* = 8.7 Hz, 1H), 7.52 (dd, *J* = 8.8, 2.4 Hz, 1H). ^{13}C NMR (100 MHz, DMSO) δ 181.4, 164.9, 150.7 (2C), 139.9, 139.8, 130.5, 130,3, 127.4, 126.2, 122.1. HRMS (ESI) m/z calculated for $C_{13}H_{11}Cl_2N_4OS$ $[M+H]^+$ 341.0025, found 341.0037. MS (ESI) *m/z*: 341 $[M+H]^+$.

2-Picolinoyl-N-(4-(trifluoromethyl)phenyl)hydrazine-1-carbothioamide (7t)

Yield 89%. ^{1}H-NMR (400 MHz, DMSO) δ 10.83 (s, 1H), 9.98 (d, *J* = 18.0 Hz, 2H), 8.74 – 8.69 (m, 1H), 8.12 – 8.03 (m, 2H), 7.79 (s, 2H), 7.68 (ddd, *J* = 7.4, 5.1, 2.7 Hz, 3H). ^{13}C-NMR (150 MHz, DMSO) δ 181.2, 166.1, 149.8, 148.9, 143.6, 138.2, 127.5 (2C), 125.9 (2C), 124.8 (q, *J* = 270 Hz), 123.0. HRMS (ESI) m/z calculated for $C_{14}H_{12}F_3N_4OS$ $[M+H]^+$ 341.0607, found 341.0600. MS (ESI) *m/z*: 341 $[M+H]^+$.

N-(4-chlorophenyl)-2-(quinoline-3-carbonyl)hydrazine-1-carbothioamide (7v)

Yield 42%. ^{1}H-NMR (400 MHz, MeOD) δ 9.36 (d, *J* = 2.2 Hz, 1H), 8.96 (d, *J* = 1.9 Hz, 1H), 8.14 (t, *J* = 8.7 Hz, 2H), 7.95 (ddd, *J* = 8.5, 6.9, 1.5 Hz, 1H), 7.76 (ddd, *J* = 8.1, 7.0, 1.1 Hz, 1H), 7.53 (d, *J* = 8.7 Hz, 2H), 7.41 – 7.33 (m, 2H). ^{13}C-NMR (100 MHz, DMSO) δ 180.8, 164.7, 149.0, 148.5, 138.2, 136.4, 135.2, 131.5, 129.2, 129.0, 128.8, 127.9, 127.5, 127.3, 126.3. HRMS (ESI) m/z calculated for $C_{17}H_{14}ClN_4OS$ $[M+H]^+$ 357.0521, found 357.0541. MS (ESI) *m/z*: 357 $[M+H]^+$.

N-(4-chlorophenyl)-2-(3-ethoxy-7-hydroxy-2-naphthoyl)hydrazine-1-carbothioamide (7w)

Yield 40%. ^{1}H NMR (400 MHz, DMSO) *δ* 11.19 (br s, 2H), 9.96 (s, 1H), 8.40 (s, 1H), 7.68 (d, *J* = 9.0 Hz, 1H), 7.58 (s, 2H), 7.39 (d, *J* = 8.6 Hz, 2H), 7.27 (s, 2H), 7.18 (d, *J* = 8.9 Hz, 1H), 4.11 (dd, *J* = 13.5, 6.6 Hz, 2H), 1.38 (t, *J* = 6.8 Hz, 3H). ^{13}C NMR (100 MHz, DMSO) δ 182.2, 161.8, 154.9, 153.1, 152.2, 140.1, 138.2, 136.2, 135.5, 131.4, 129.0, 128.1, 127.4, 121.5, 110.7, 107.2, 63.1, 14.6. HRMS (ESI) m/z calculated for $C_{20}H_{19}ClN_3O_3S$ $[M+H]^+$ 416.0830, found 416.0849. MS (ESI) *m/z*: 416 $[M+H]^+$.

N-(4-chlorophenyl)-2-(3,5-dihydroxy-2-naphthoyl)hydrazine-1-carbothioamide (7x)

Yield 42%. ^{1}H NMR (400 MHz, DMSO) δ 11.39 (s, 1H), 10.76 (s, 1H), 10.09 (s, 1H), 9.91 (s, 1H), 8.45 (s, 1H), 7.56 (s, 2H), 7.53 (s, 1H), 7.44 – 7.38 (m, 2H), 7.36 (d, *J* = 8.2 Hz, 1H), 7.20 – 7.13 (m, 1H), 6.88 (d, *J* = 6.9 Hz, 1H). ^{13}C NMR (100 MHz, DMSO) δ 179.1, 163.2, 153.4, 151.6, 138.1, 134.3, 130.4, 128.1, 127.7, 126.9, 126.7, 124.2, 119.3, 118.9, 109.7, 105.6. HRMS (ESI) m/z calculated for $C_{18}H_{15}ClN_3O_3S$ $[M+H]^+$ 388.0517, found 388.0531. MS (ESI) *m/z*: 388 $[M+H]^+$.

N-(4-chlorophenyl)-2-(4-ethoxy-1-hydroxy-2-naphthoyl)hydrazine-1-carbothioamide (7y)

Yield 37%. ^{1}H NMR (400 MHz, DMSO) *δ* 13.32 (s, 1H), 10.99 (s, 1H), 10.02 (s, 1H), 9.97 (s, 1H), 8.29 (dd, J = 8.2, 0.6 Hz, 1H), 8.15 (d, J = 7.8 Hz, 1H), 7.66 (dddd, J = 26.8, 8.2, 6.9, 1.3 Hz, 2H), 7.50 (s, 2H), 7.40 (d, J = 8.8 Hz, 2H), 7.34 (s, 1H), 4.22 (q, J = 7.0 Hz, 2H), 1.49 (t, J = 7.0 Hz, 3H). ^{13}C NMR (100 MHz, DMSO) δ 181.7, 171.0, 154.3, 146.4, 138.6, 129.3, 128.9, 128.4 (2C), 128.1, 127.0, 125.7, 123.6, 122.1, 105.7, 101.9, 64.5, 15.2. HRMS (ESI) m/z calculated for $C_{20}H_{19}ClN_3O_3S$ $[M+H]^+$ 416.0830, found 416.0846. m/z: 416 [M+H]+. MS (ESI) *m/z*: 416 $[M+H]^+$.

N-(4-chlorophenyl)-2-(pyridin-2-ylmethyl)hydrazine-1-carbothioamide (12)

1-Chloro-4-isothiocyanatobenzene (11, 229 mg, 1.35 mmol) was added to a solution of **10** (111 mg, 0.9 mmol) in EtOH (8 mL). The mixture was stirred at rt for 4 h then, the reaction mixture was concentrated, diluted with EA, washed with water, brine, and dried over anhydrous $MgSO_4$. The residue was purified by using silica gel chromatography (20% EA in Hexanes) to give **12** as a yellow solid (53 mg, 20%). ^{1}H NMR (400 MHz, DMSO) *δ* 10.35 (s, 1H), 8.54 (ddd, *J* = 4.8, 1.7, 1.0 Hz, 1H), 7.80 (td, *J* = 7.7, 1.8 Hz, 1H), 7.68 – 7.61 (m, 2H), 7.37 – 7.34 (m, 2H), 7.33 – 7.28 (m, 2H), 5.43 (s, 2H), 5.26 (s, 2H). ^{13}C-NMR (100 MHz, DMSO) δ 180.3, 157.1, 149.5, 139.4, 137.3, 128.6, 128.3, 126.3, 122.8, 122.1, 58.4. HRMS (ESI) m/z calculated for $C_{13}H_{14}ClN_4S$ $[M+H]^+$ 293.0570, found 293.0596. MS (ESI) *m/z*: 293 $[M+H]^+$.

N-(4-chlorophenyl)-1-methyl-2-picolinoylhydrazine-1-carbothioamide (14)

Yield 31%. ^{1}H-NMR (400 MHz, DMSO) δ 11.21 (s, 1H), 9.79 (s, 1H), 8.72 (ddd, *J* = 4.7, 1.6, 1.0 Hz, 1H), 8.13 – 8.00 (m, 2H), 7.68 (ddd, *J* = 7.3, 4.8, 1.6 Hz, 1H), 7.37 (s, 4H), 3.55 (s, 3H). ^{13}C-NMR (175 MHz, MeOD + DMSO) δ 181.21 (s), 163.1, 149.3, 148.4, 139.1, 137.6, 128.9, 127.9, 127.7, 127.1, 122.6, 41.0. HRMS (ESI) m/z calculated for $C_{14}H_{14}ClN_4OS$ $[M+H]^+$ 321.0520, found 321.0563. MS (ESI) *m/z*: 321 $[M+H]^+$.

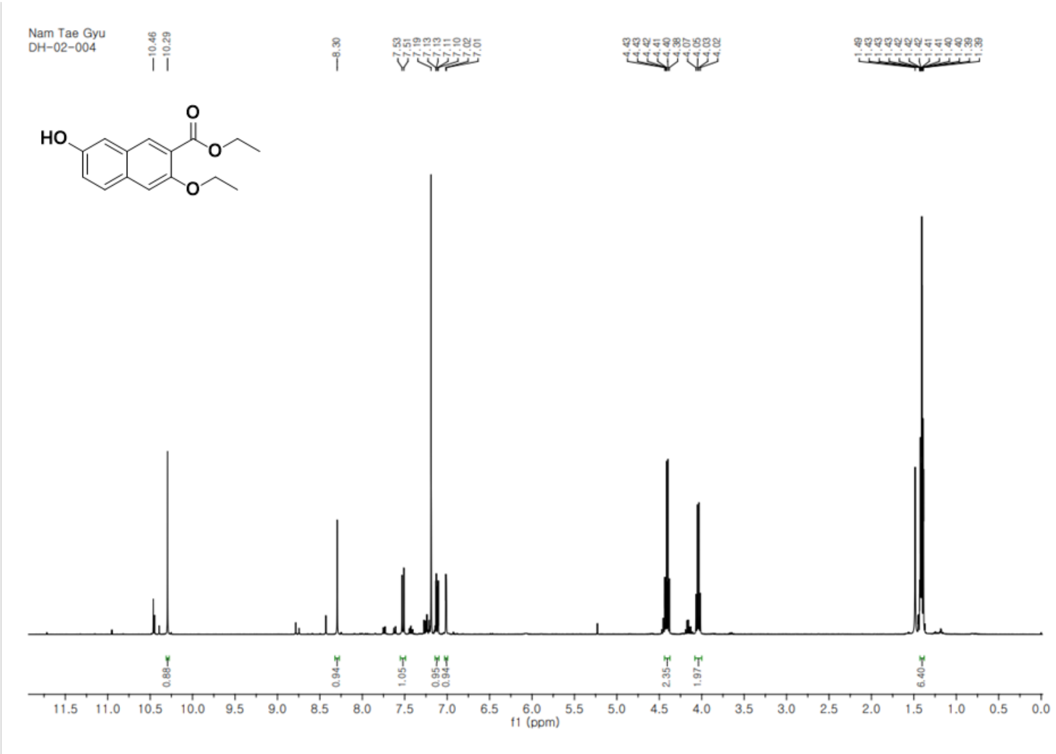

^{1}H-NMR spectrum of compound **2w**

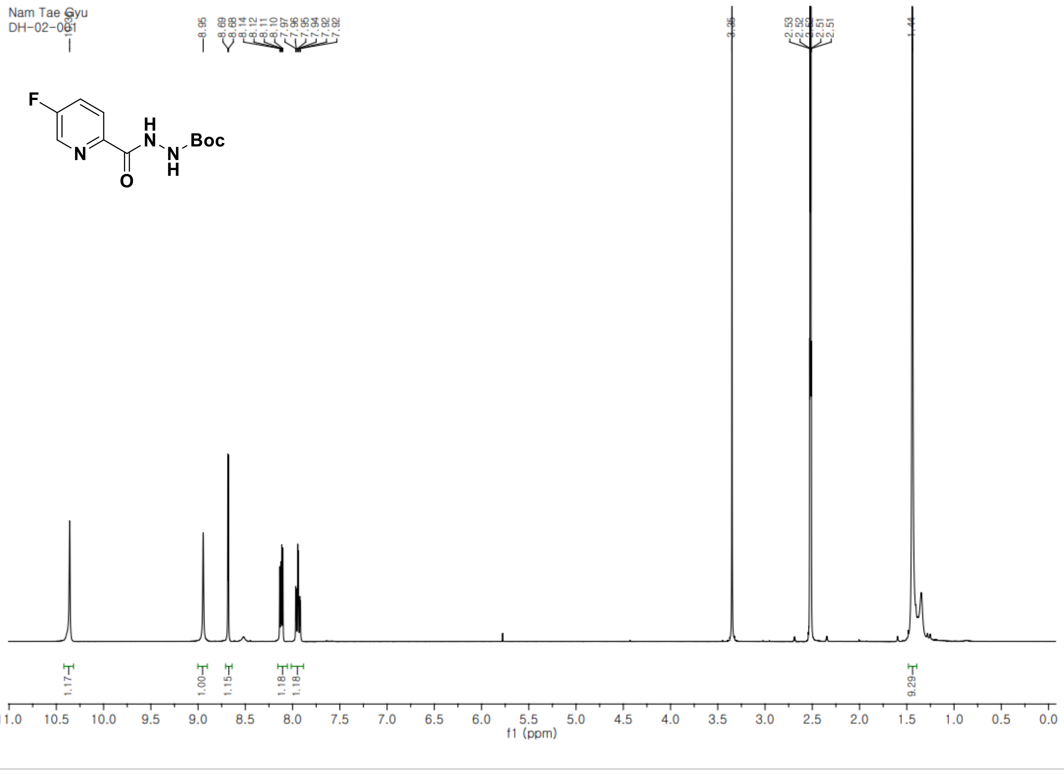

^{1}H-NMR spectrum of compound **3k**

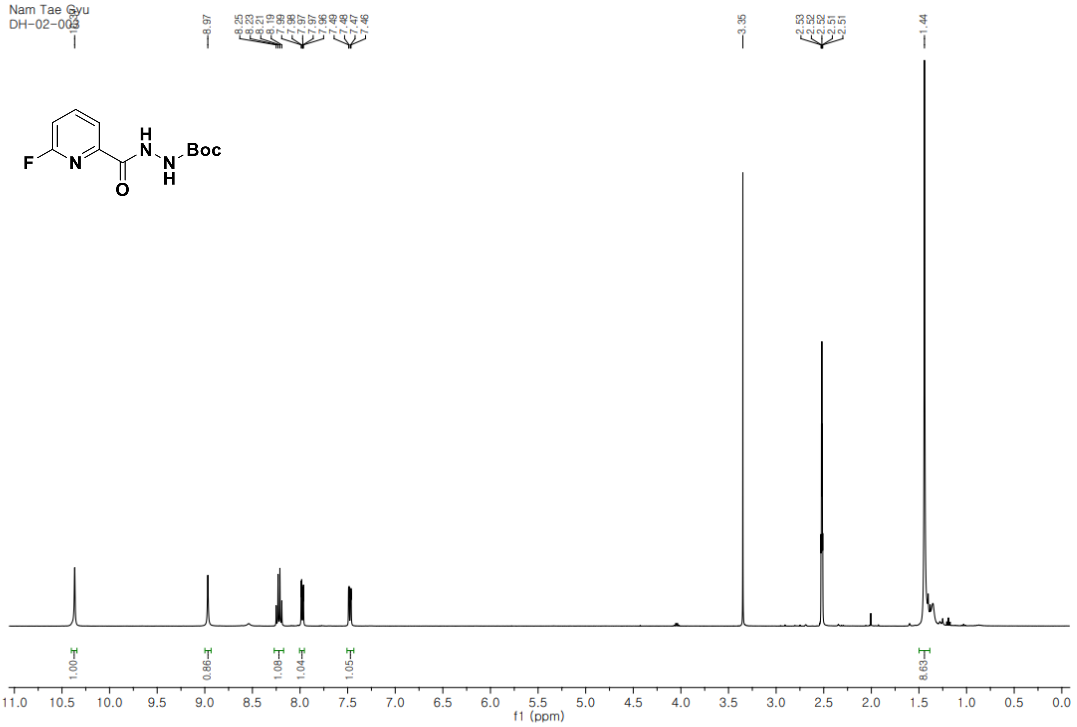

^{1}H-NMR spectrum of compound **3l**

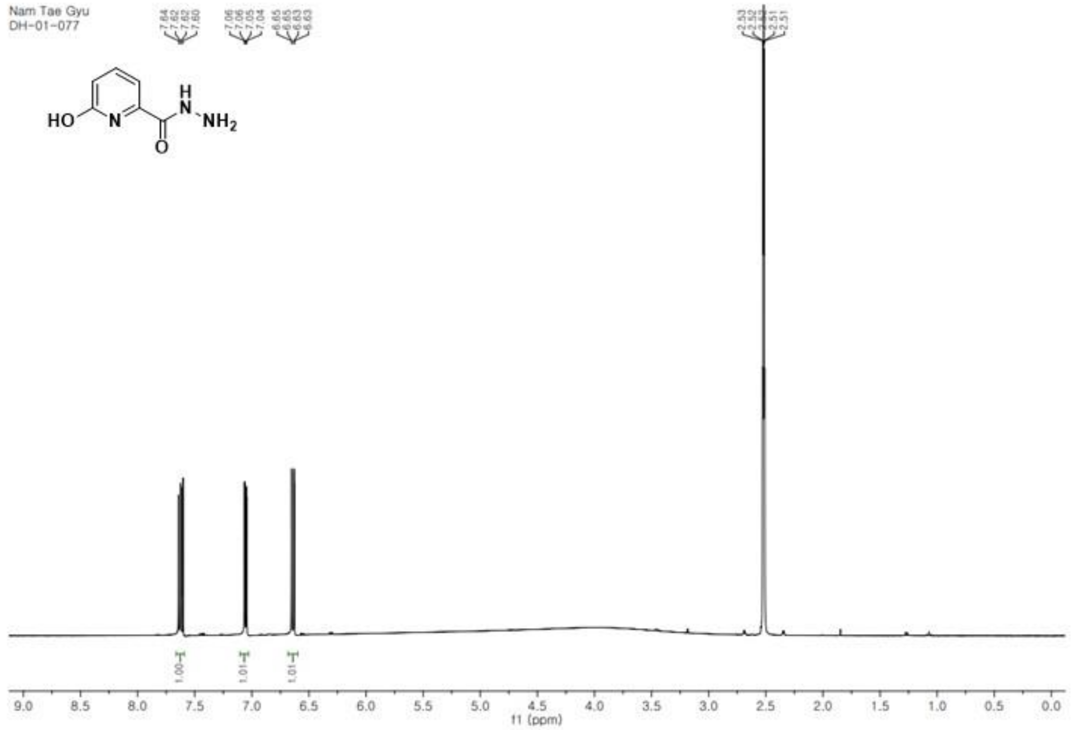

^{1}H-NMR spectrum of compound **4i**

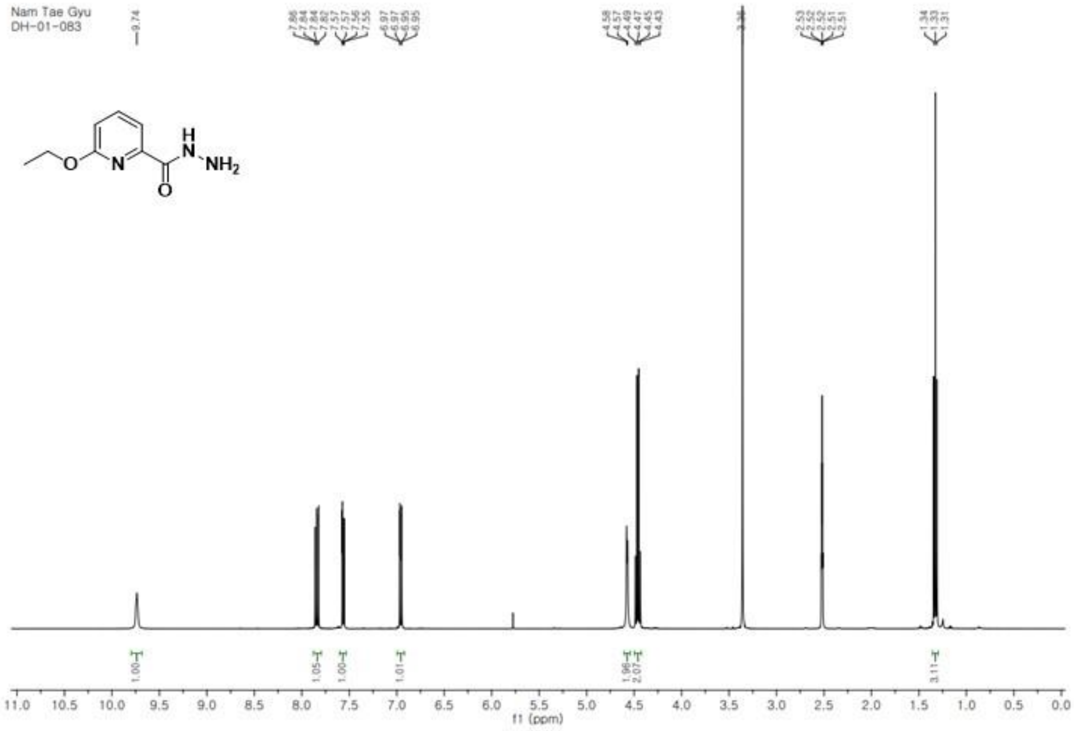

^{1}H-NMR spectrum of compound **4j**

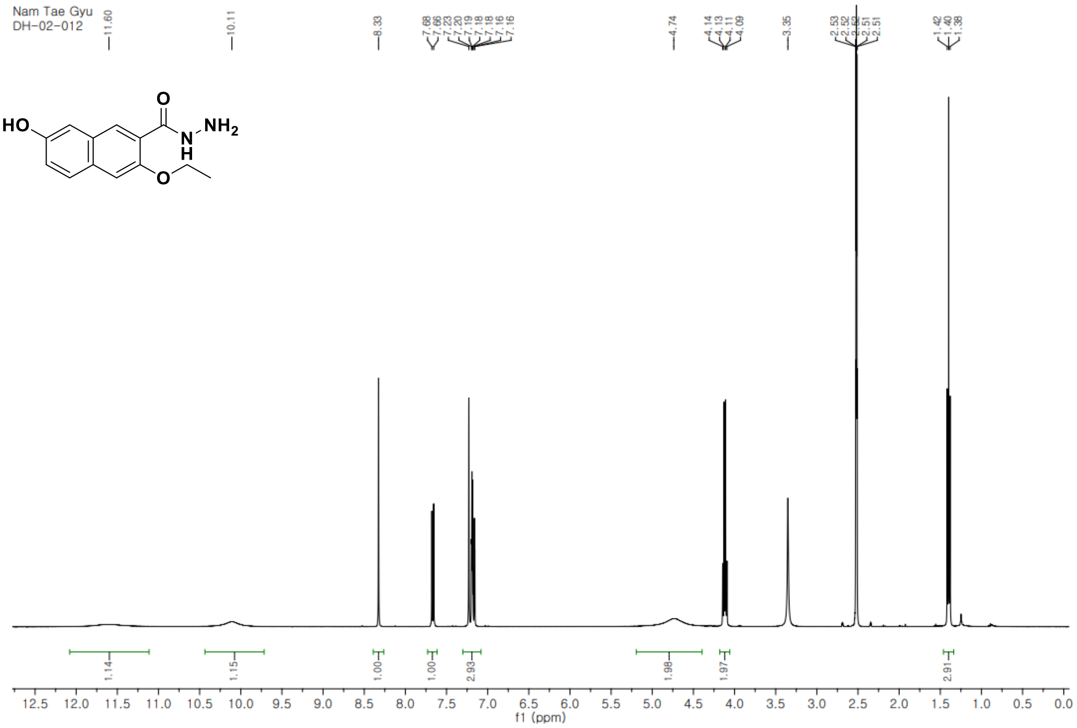

^{1}H-NMR spectrum of compound **4w**

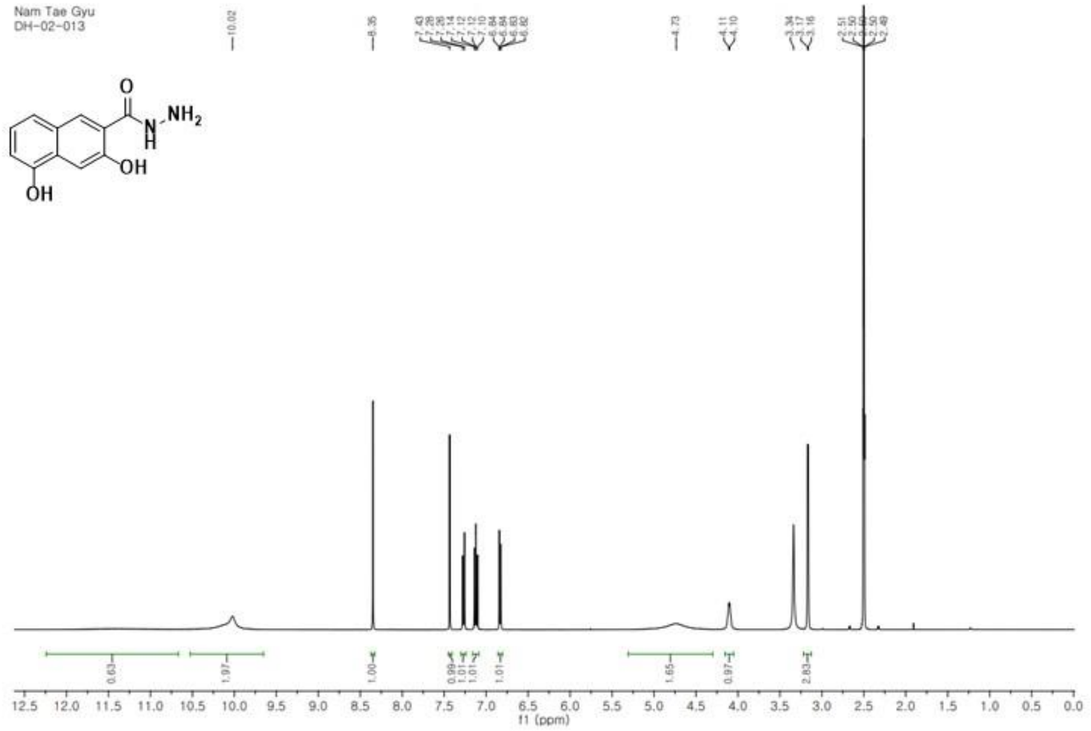

^{1}H-NMR spectrum of compound **4x**

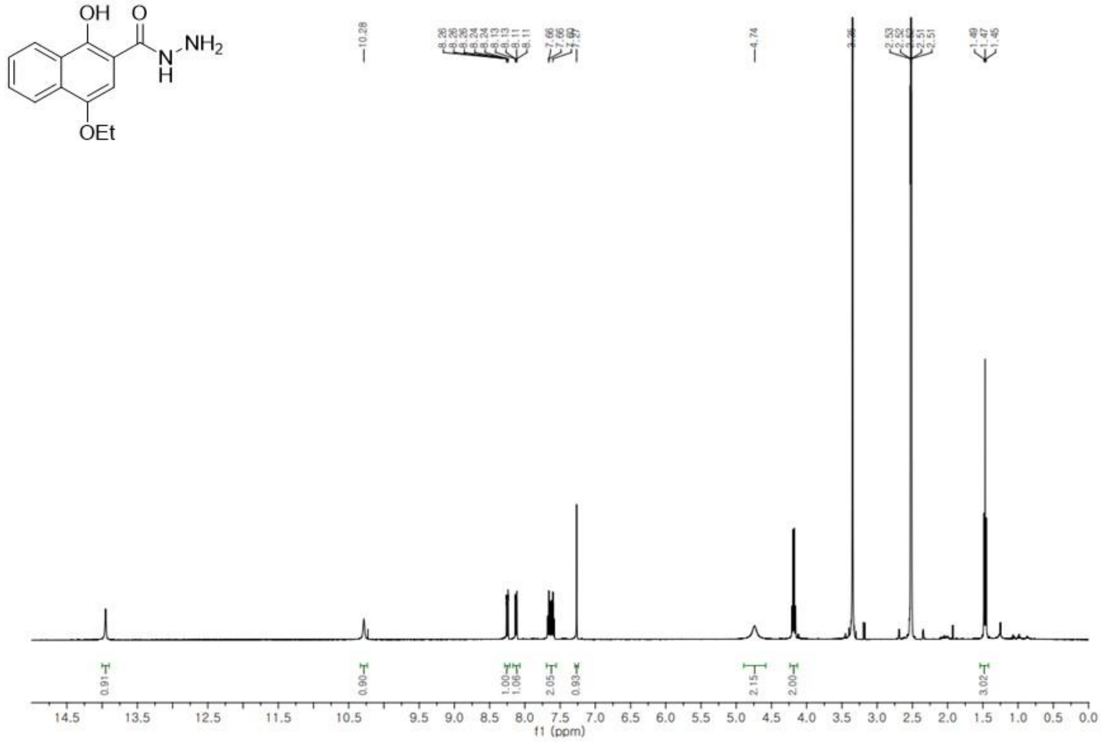

^{1}H-NMR spectrum of compound **4y**

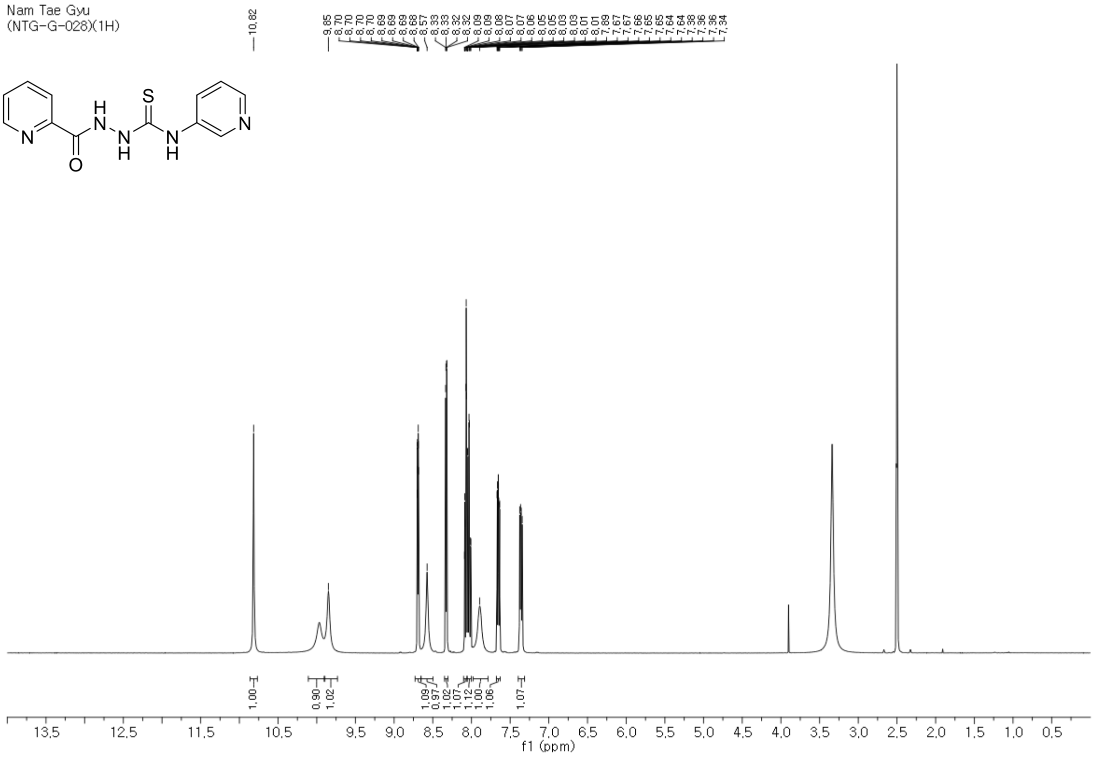

^{1}H-NMR spectrum of Compound **7d**

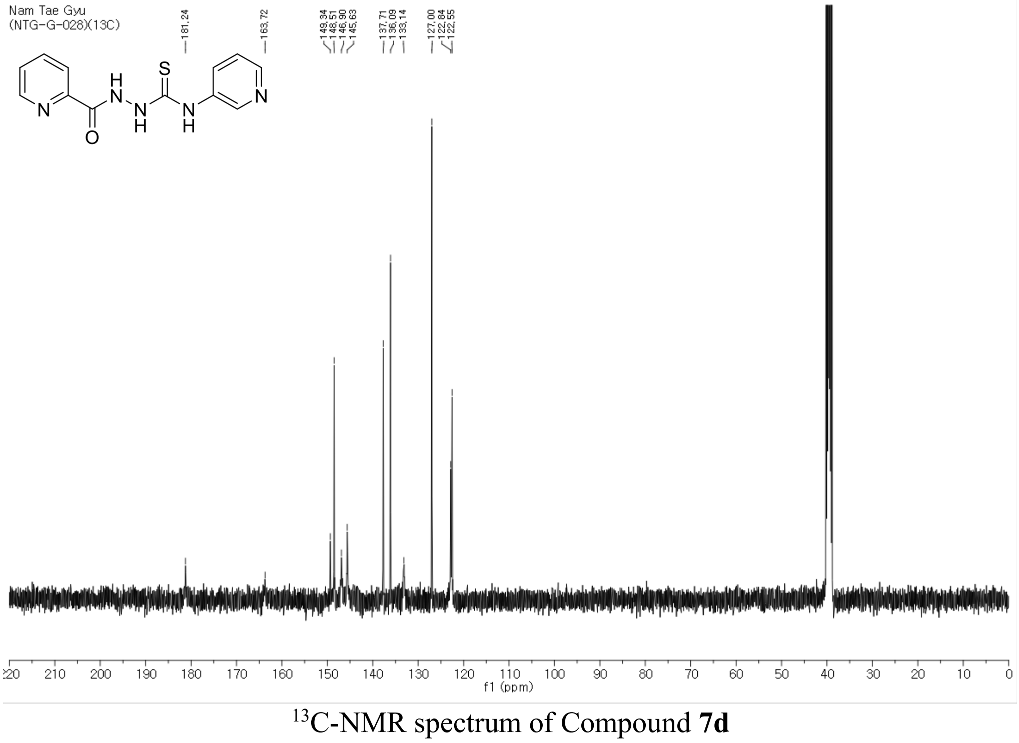

^{13}C-NMR spectrum of Compound **7d**

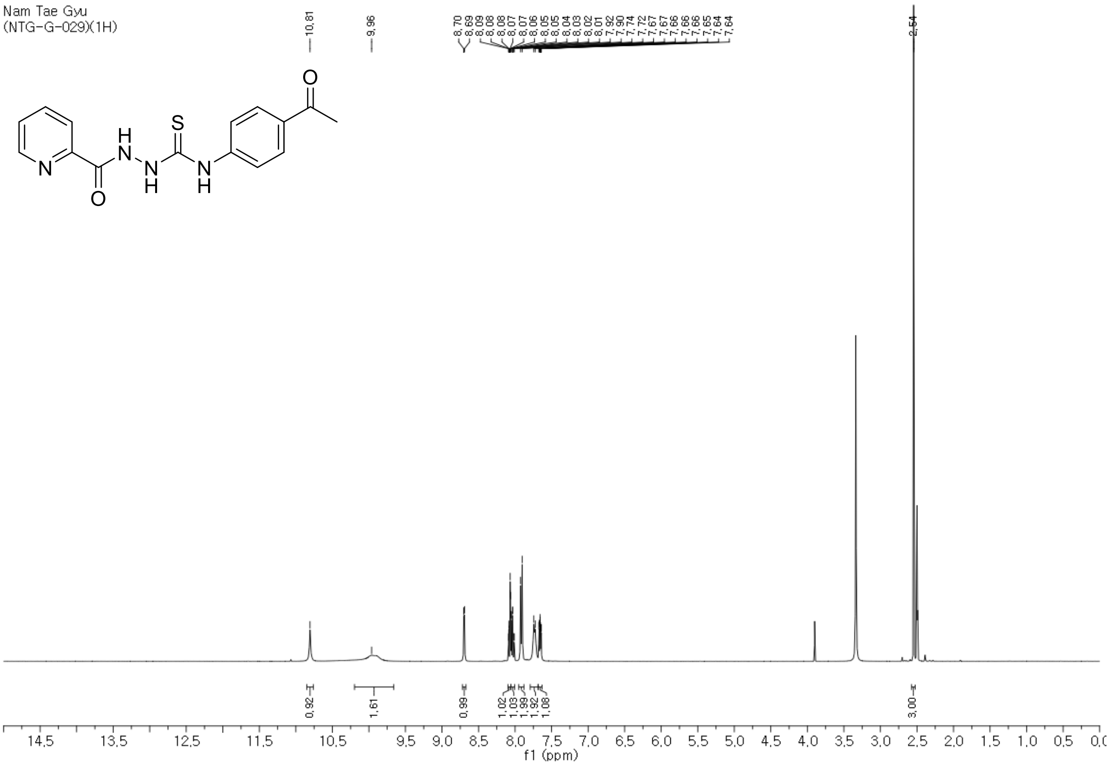

^{1}H-NMR spectrum of Compound **7e**

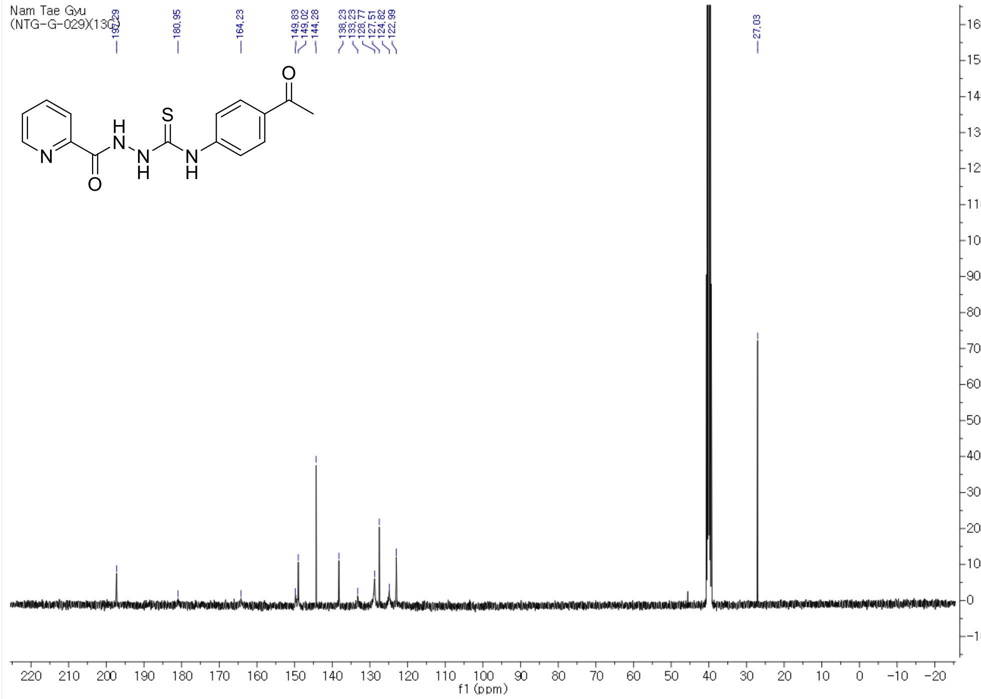

^{13}C-NMR spectrum of Compound **7e**

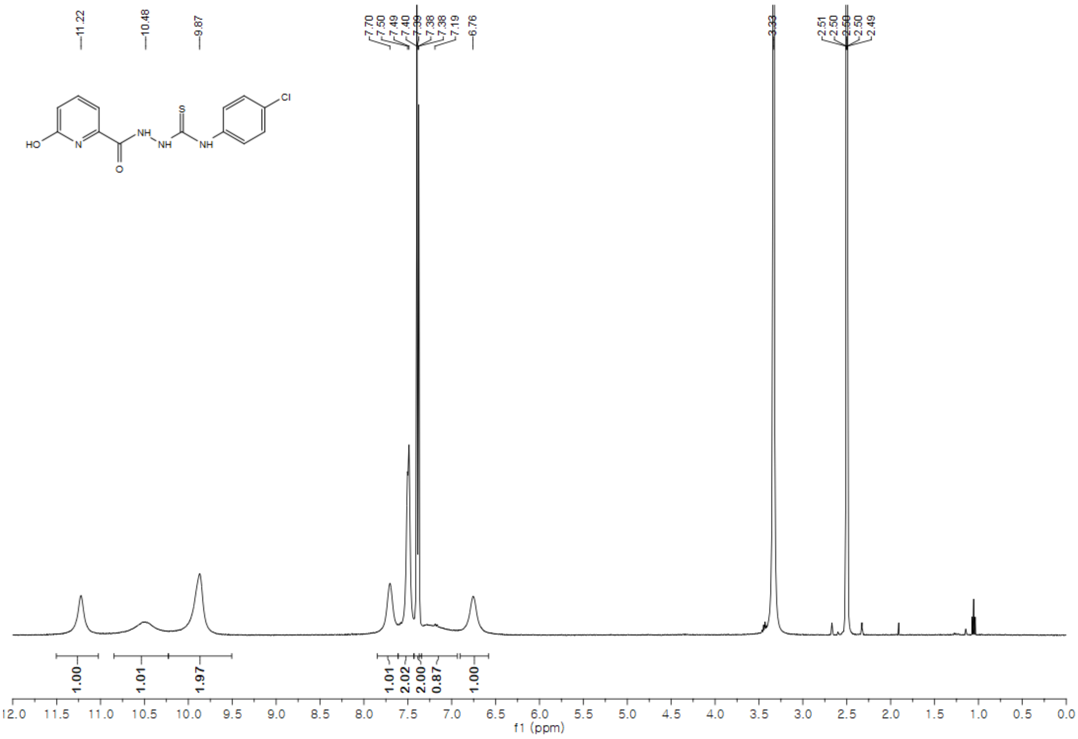

^{1}H-NMR spectrum of Compound **7i**

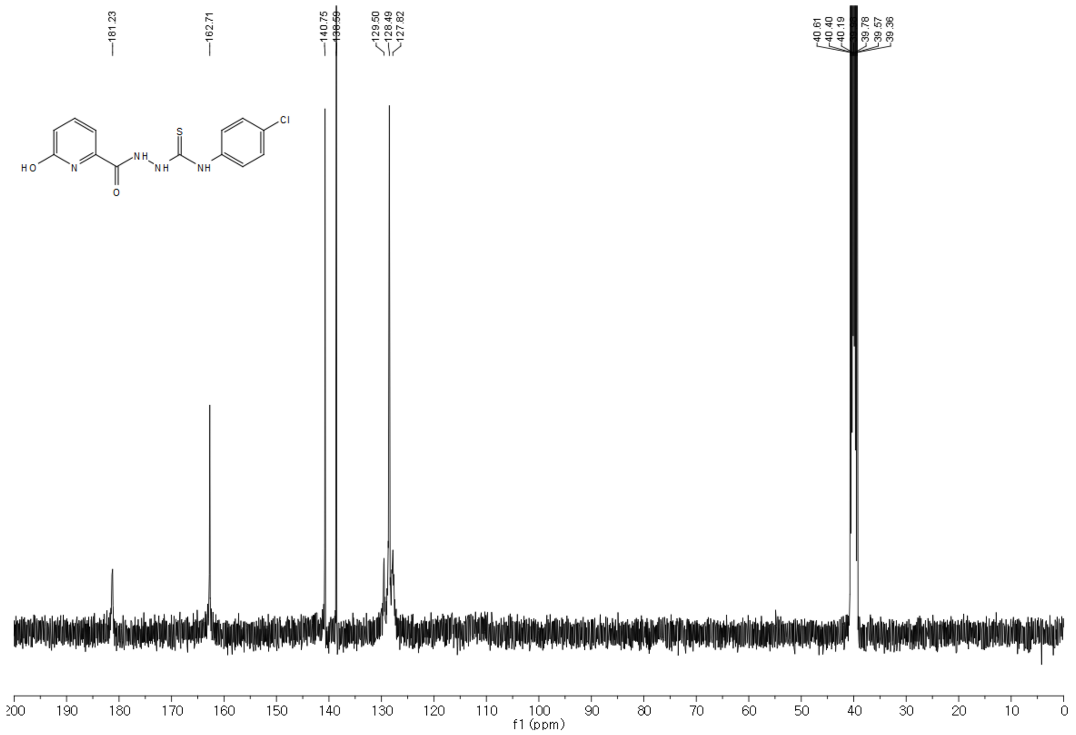

^{13}C-NMR spectrum of Compound **7i**

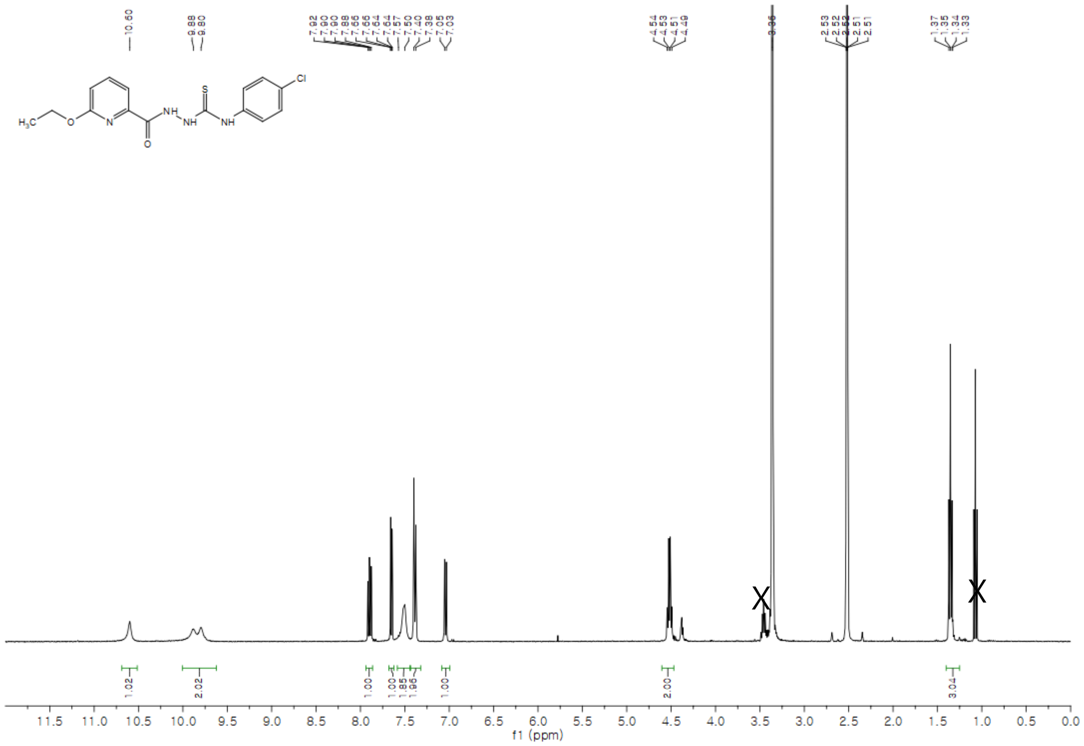

^{1}H-NMR spectrum of Compound **7j**

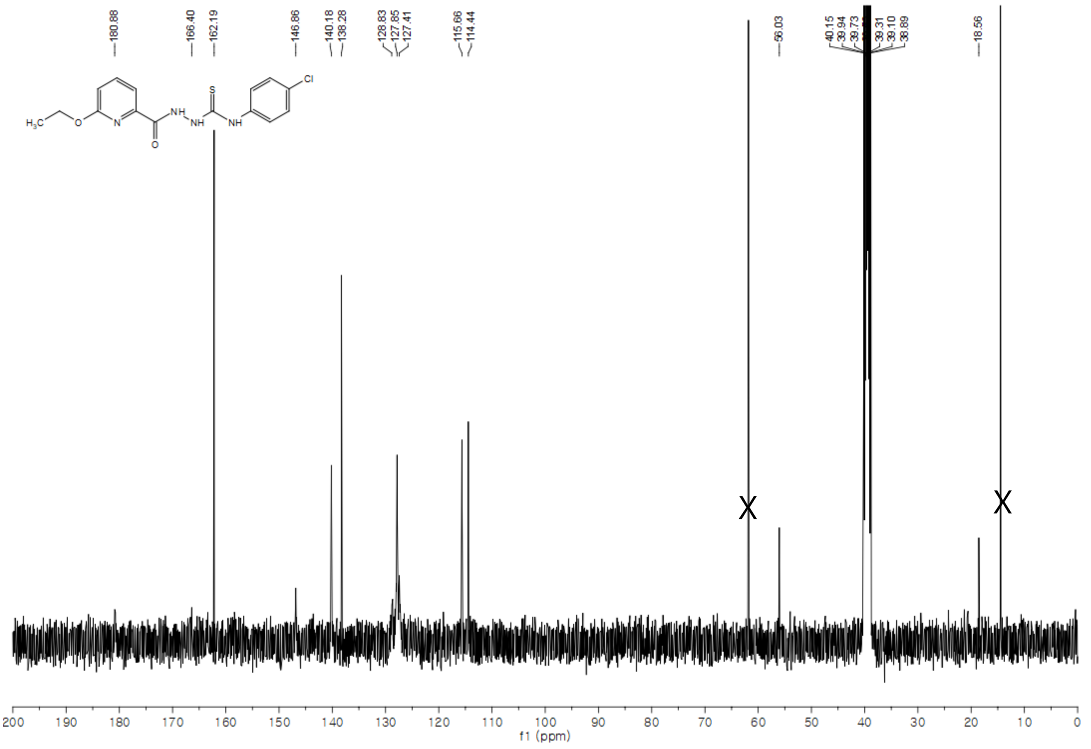

^{13}C-NMR spectrum of Compound **7j**

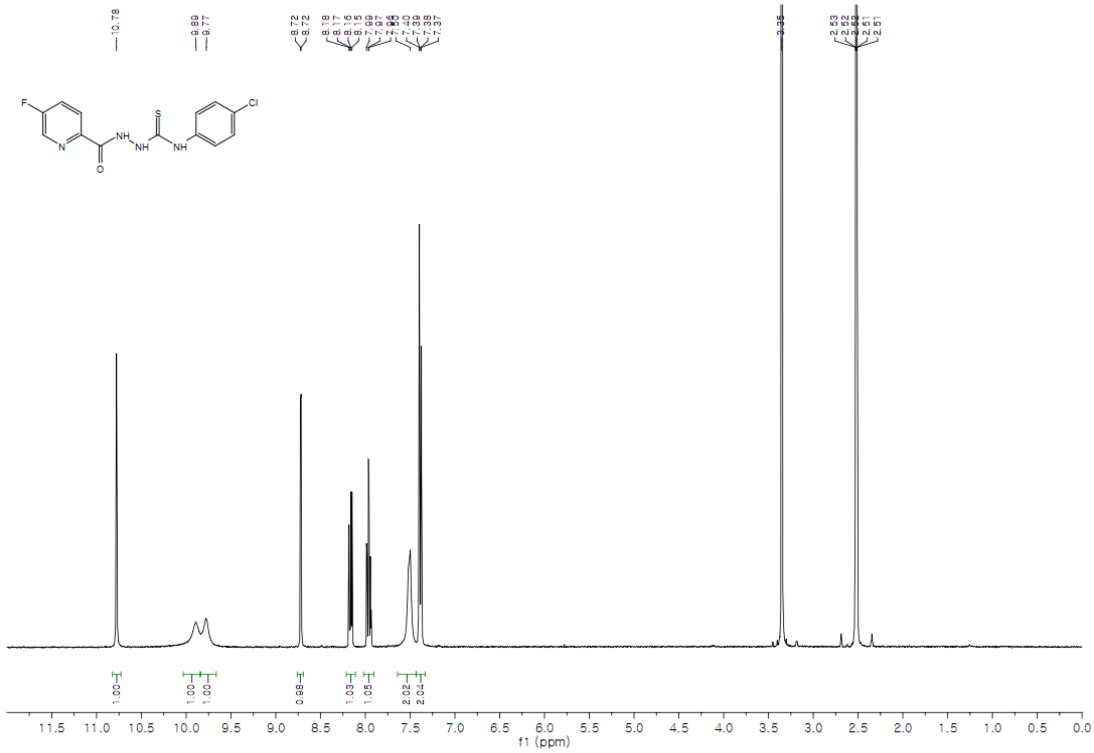

^{1}H-NMR spectrum of Compound **7k**

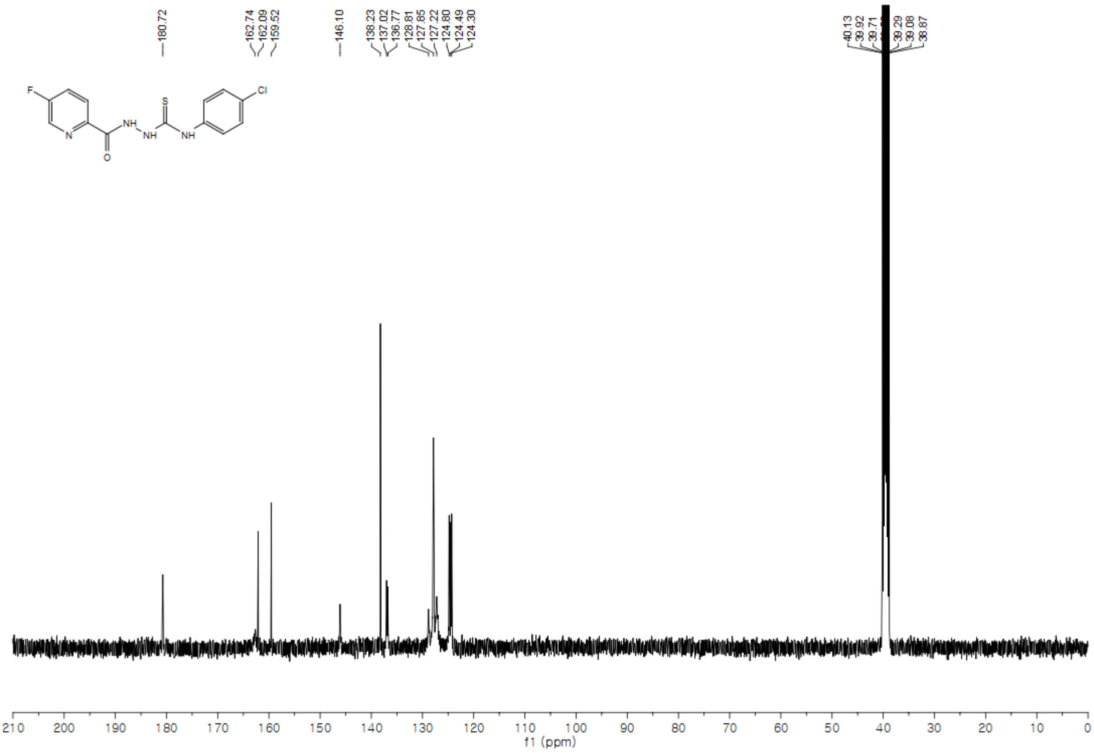

^{13}C-NMR spectrum of Compound **7k**

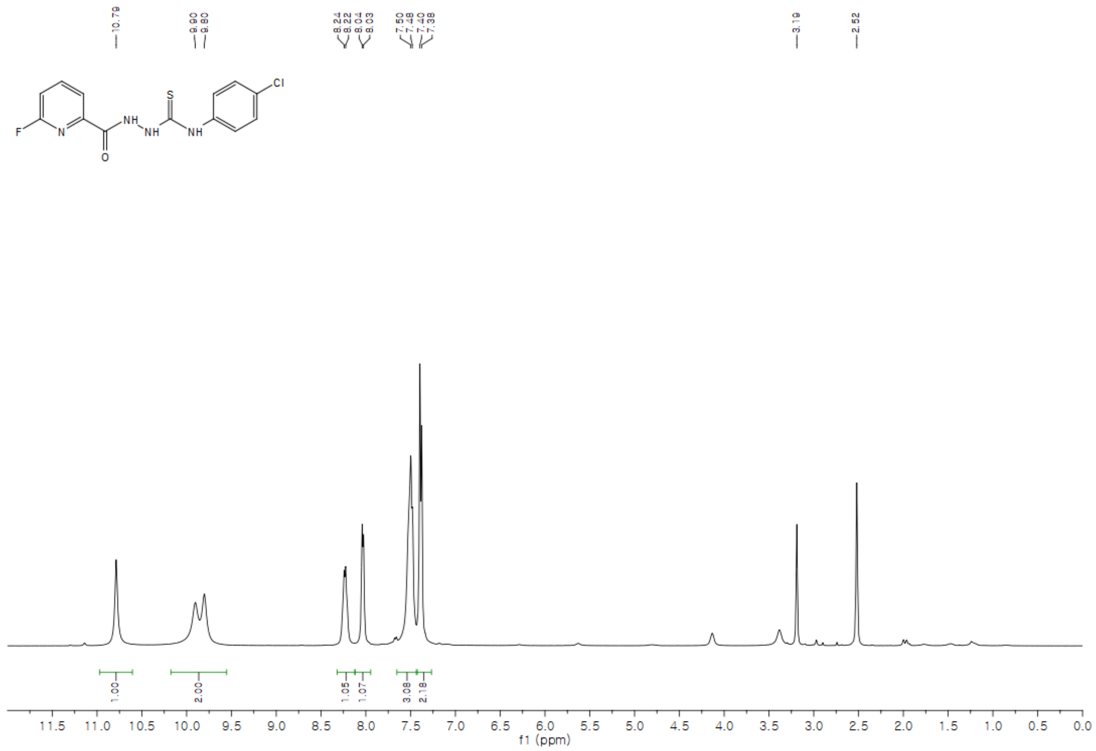

^{1}H-NMR spectrum of Compound **7l**

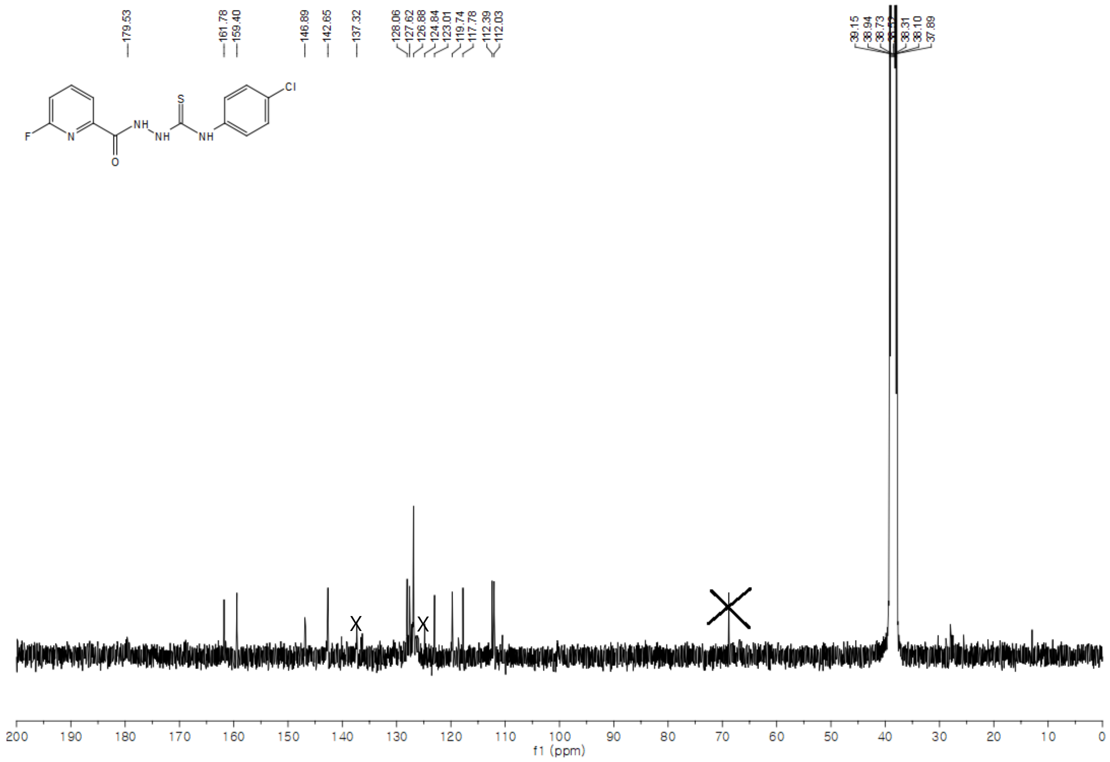

^{13}C-NMR spectrum of Compound **7l**

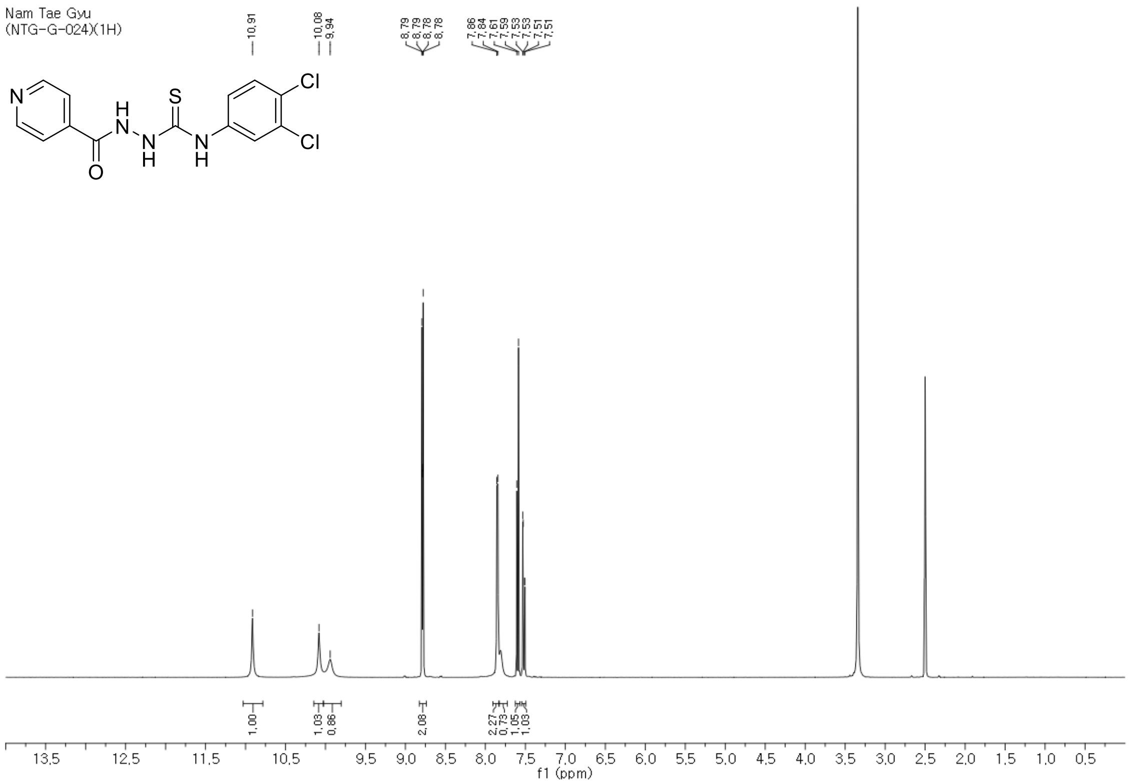

^{1}H-NMR spectrum of Compound **7r**

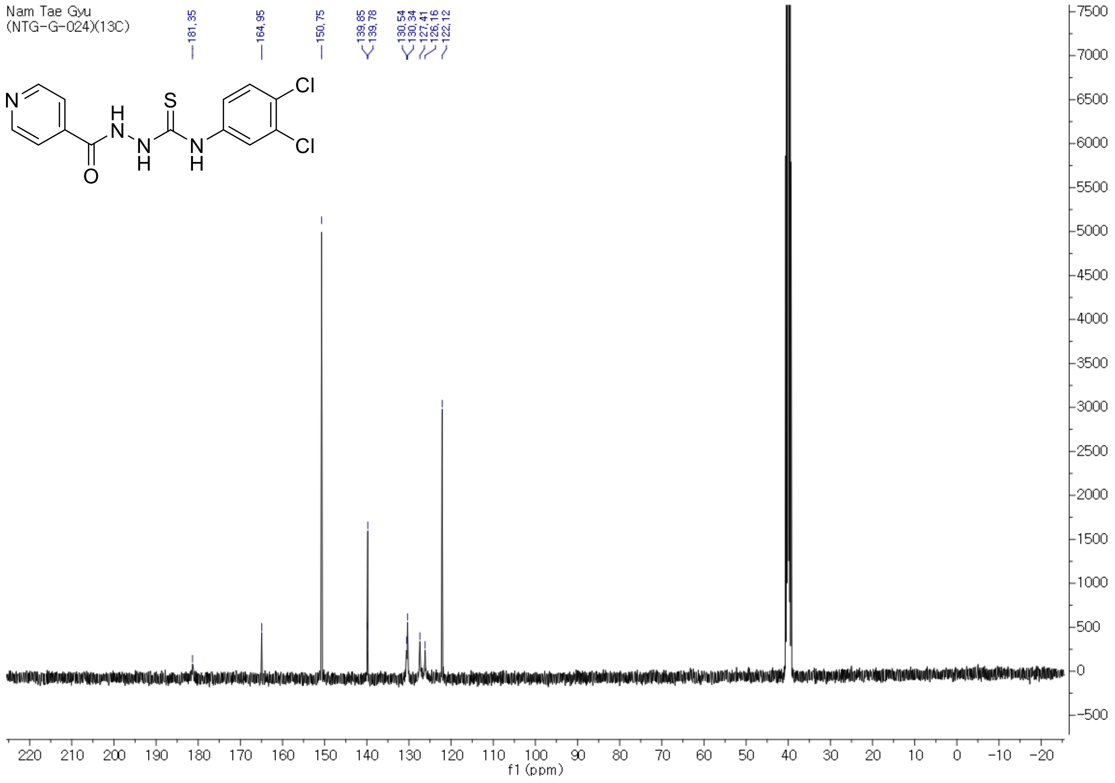

^{13}C-NMR spectrum of Compound **7r**

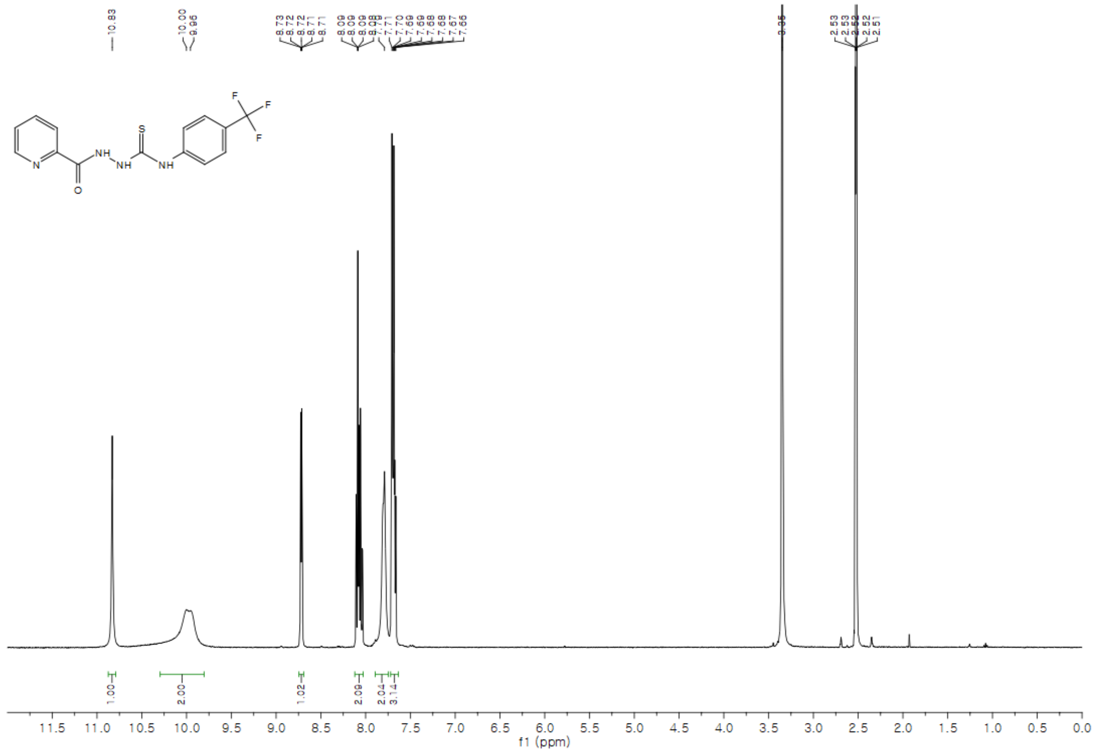

^{1}H-NMR spectrum of Compound **7t**

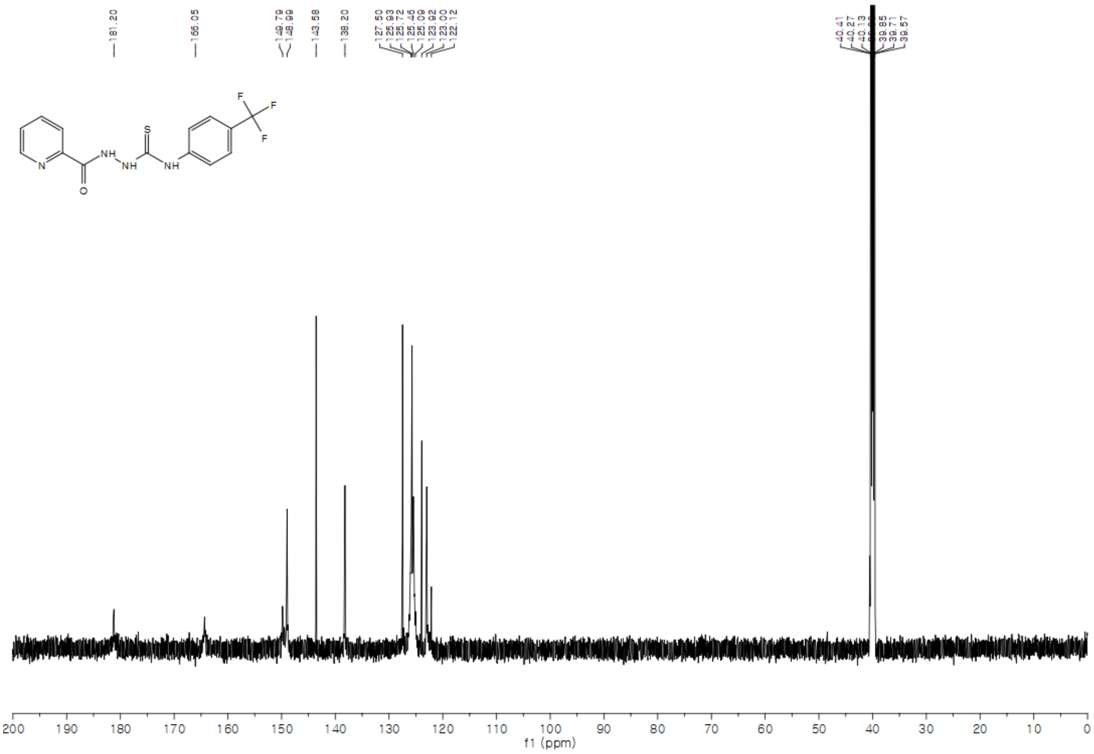

^{13}C-NMR spectrum of Compound **7t**

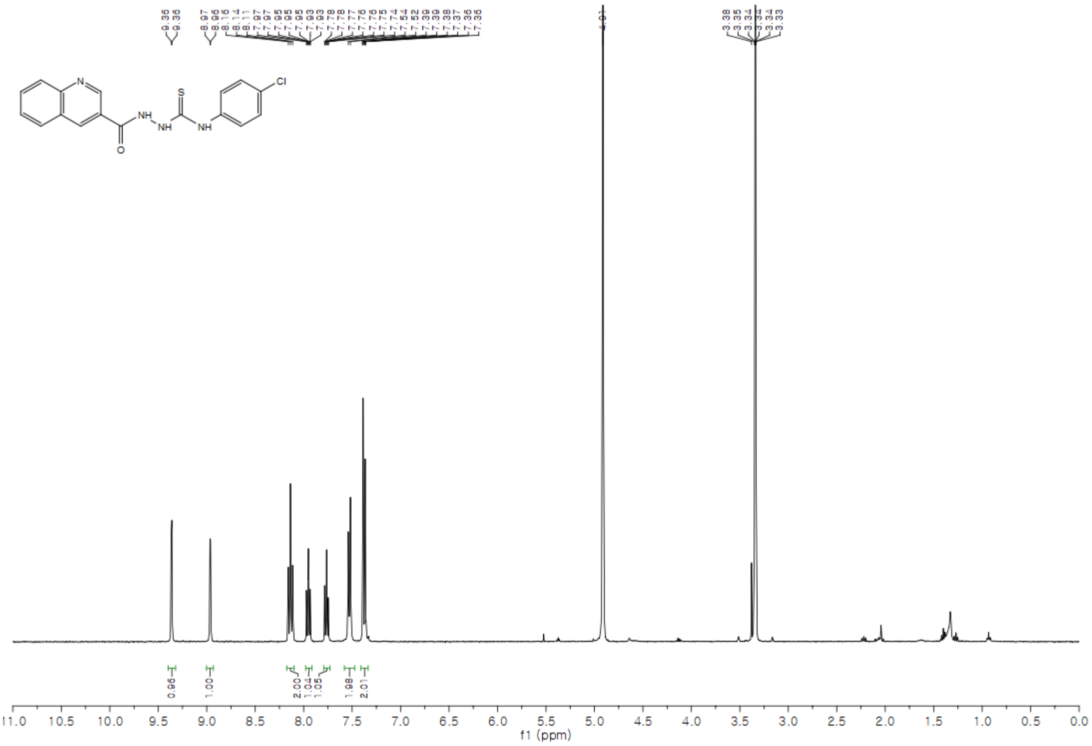

^{1}H-NMR spectrum of Compound **7v**

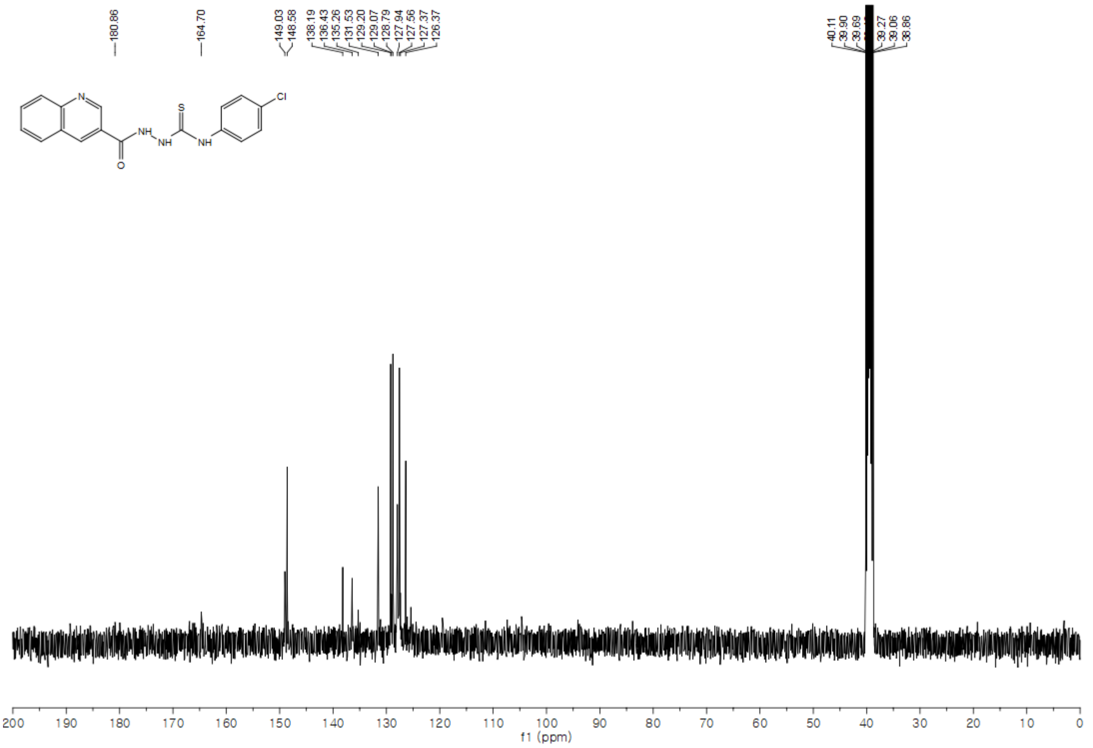

^{13}C-NMR spectrum of Compound **7v**

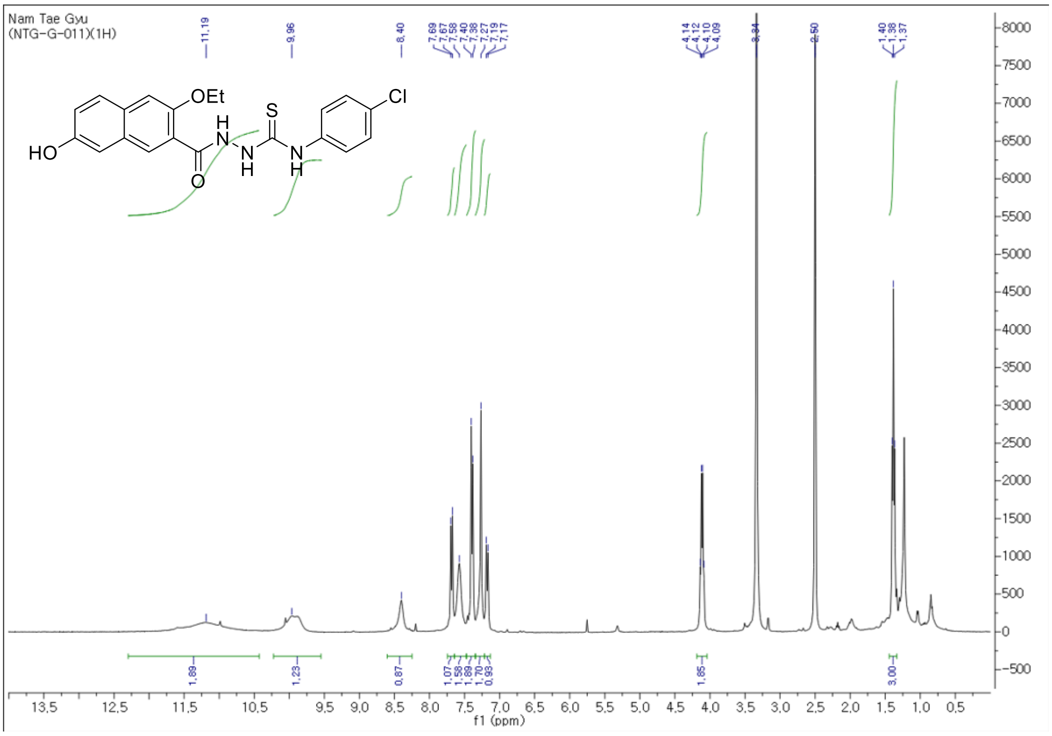

^{1}H-NMR spectrum of Compound **7w**

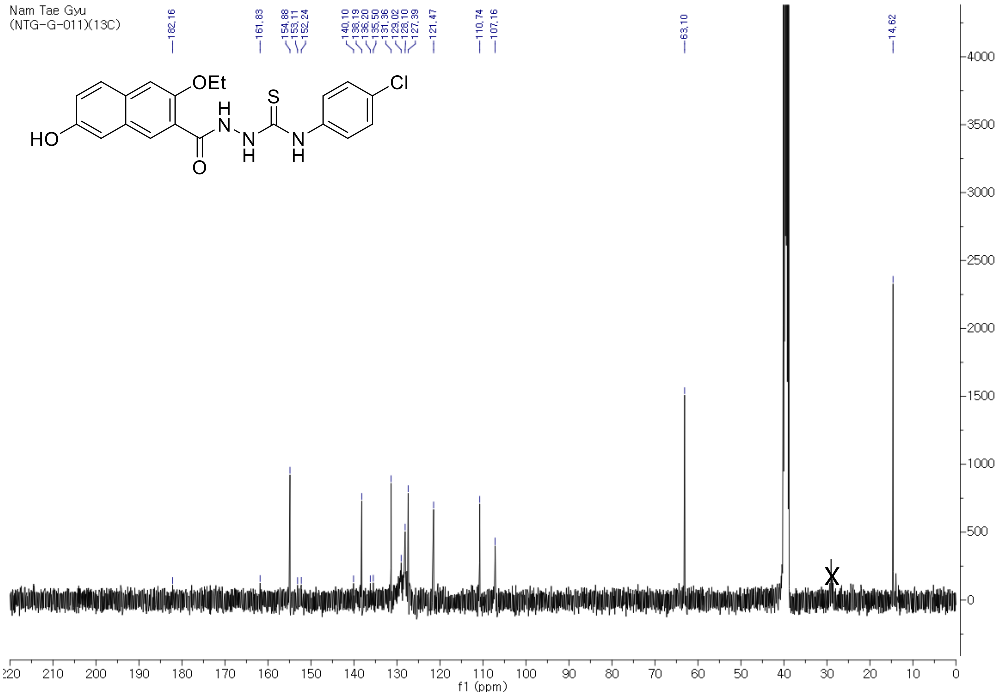

^{13}C-NMR spectrum of Compound **7w**

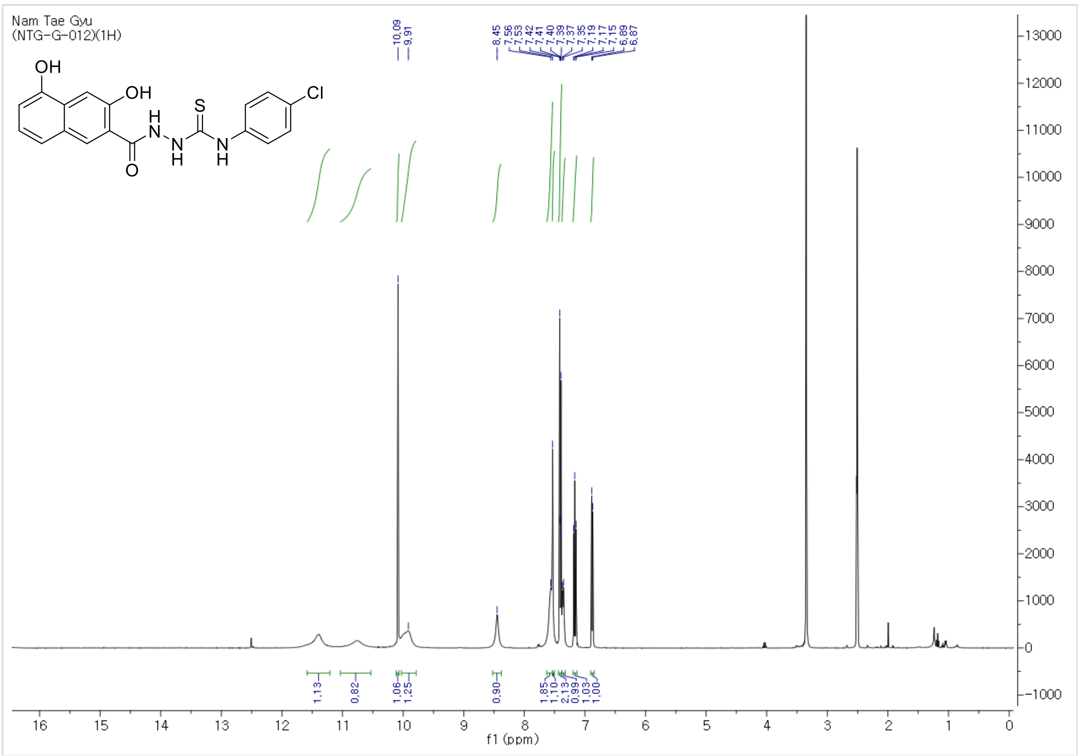

^{1}H-NMR spectrum of Compound **7x**

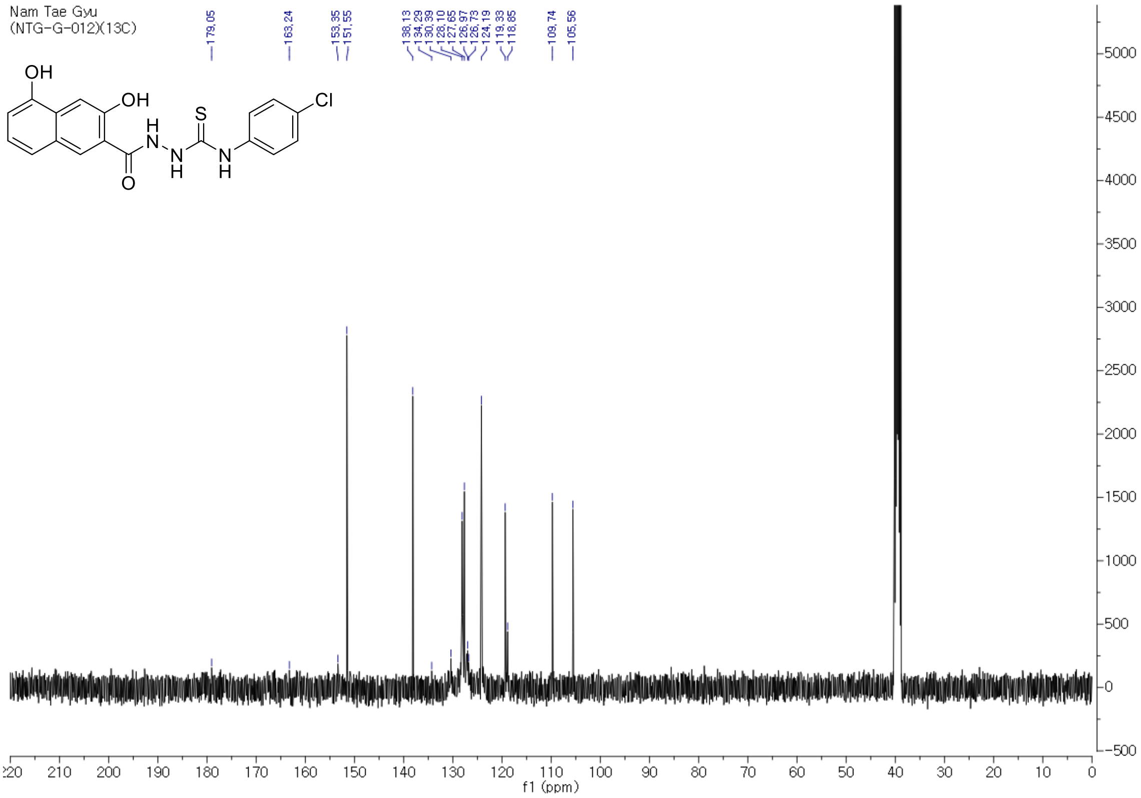

^{13}C-NMR spectrum of Compound **7x**

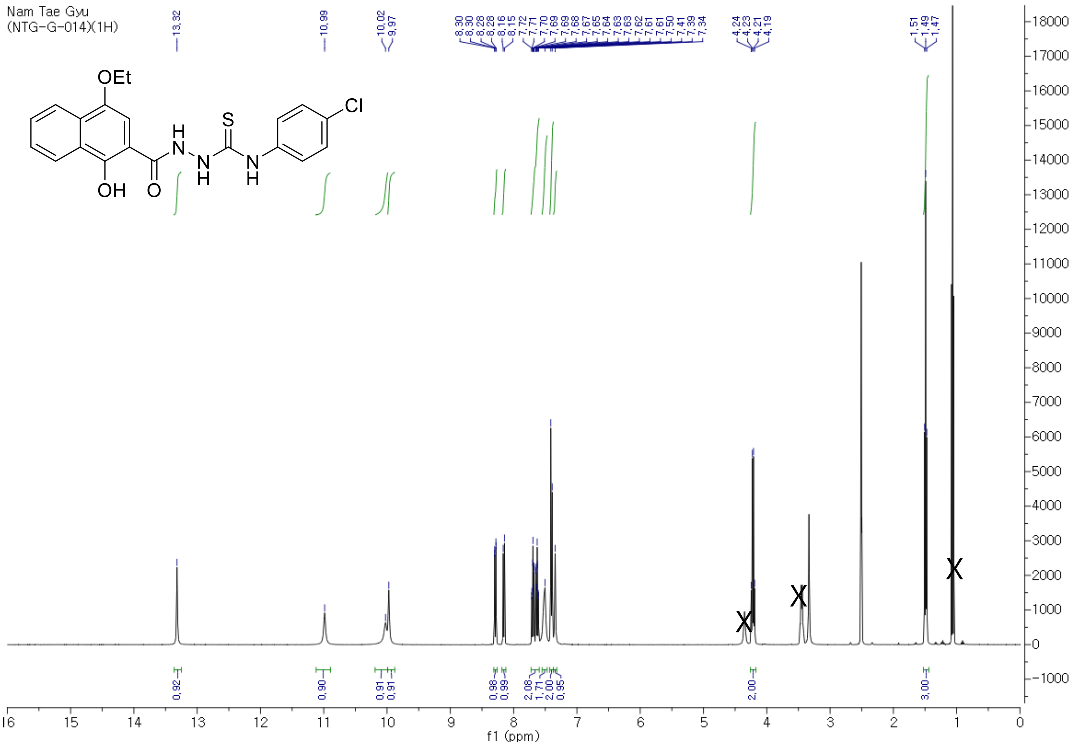

^{1}H-NMR spectrum of Compound **7y**

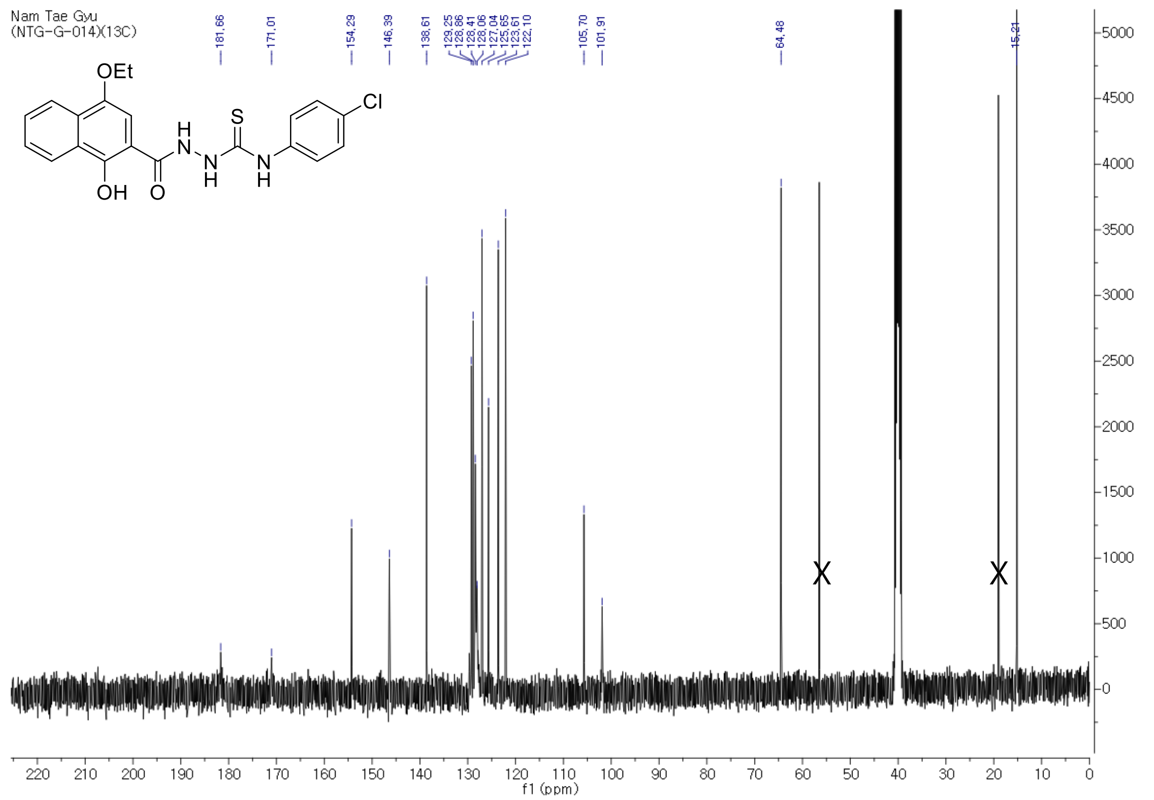

^{13}C-NMR spectrum of Compound **7y**

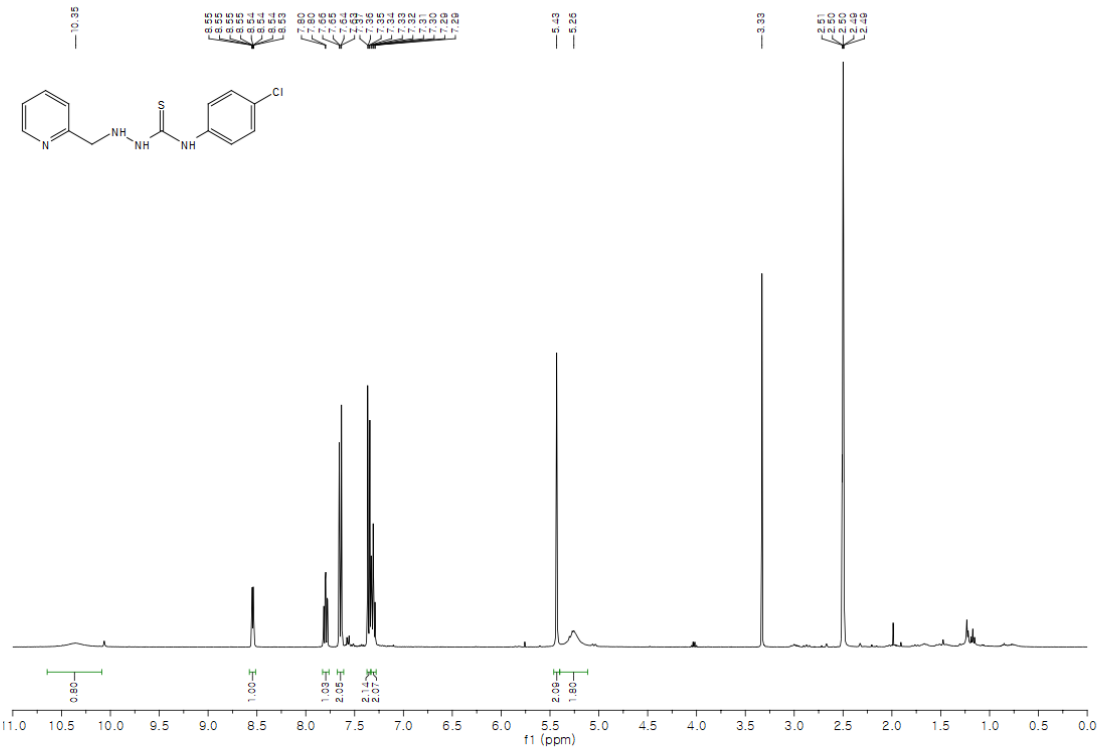

^{1}H-NMR spectrum of Compound **12**

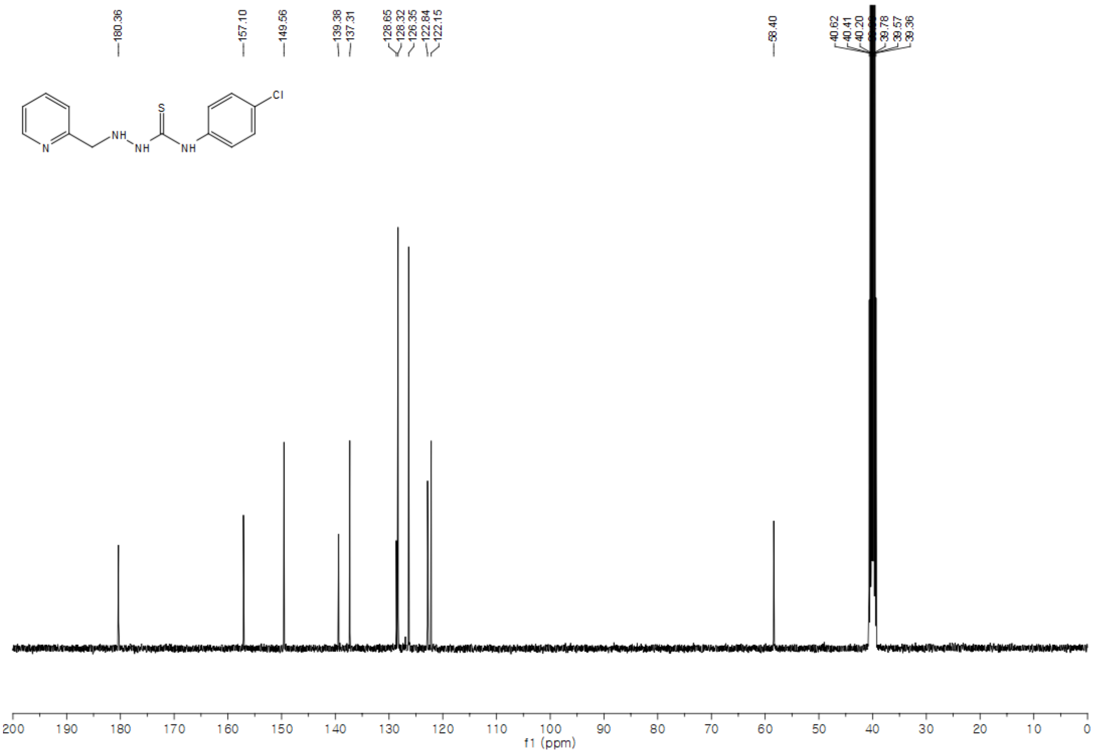

^{13}C-NMR spectrum of Compound **12**

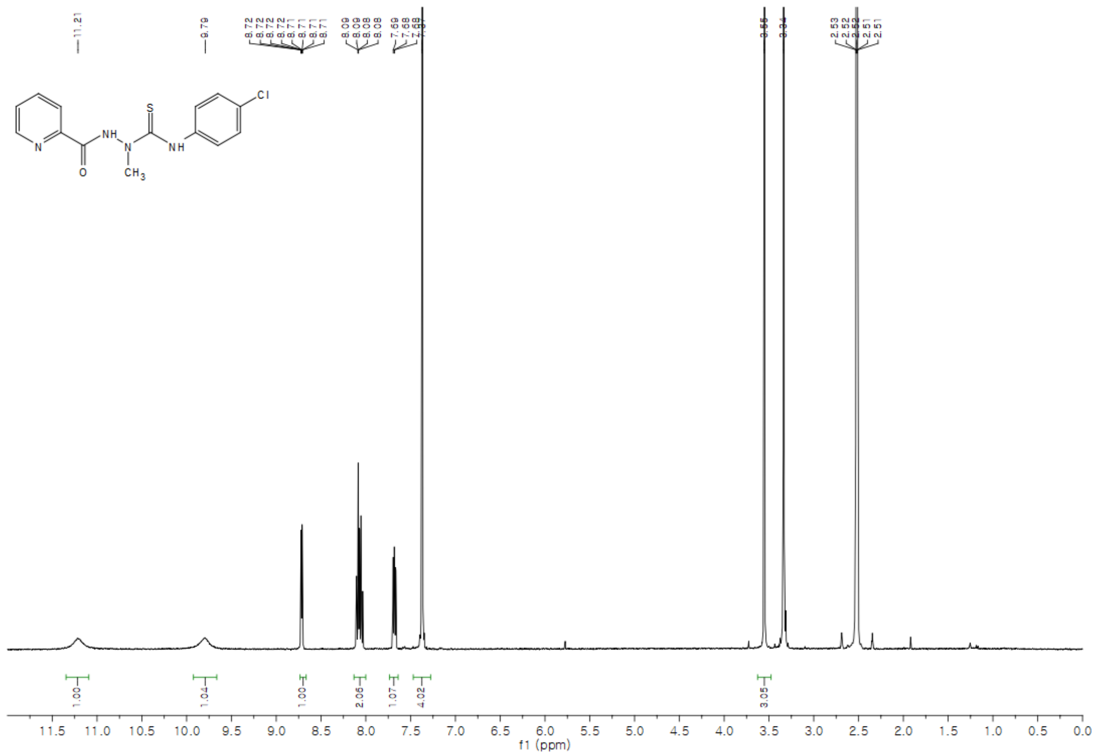

^{1}H-NMR spectrum of Compound **14**

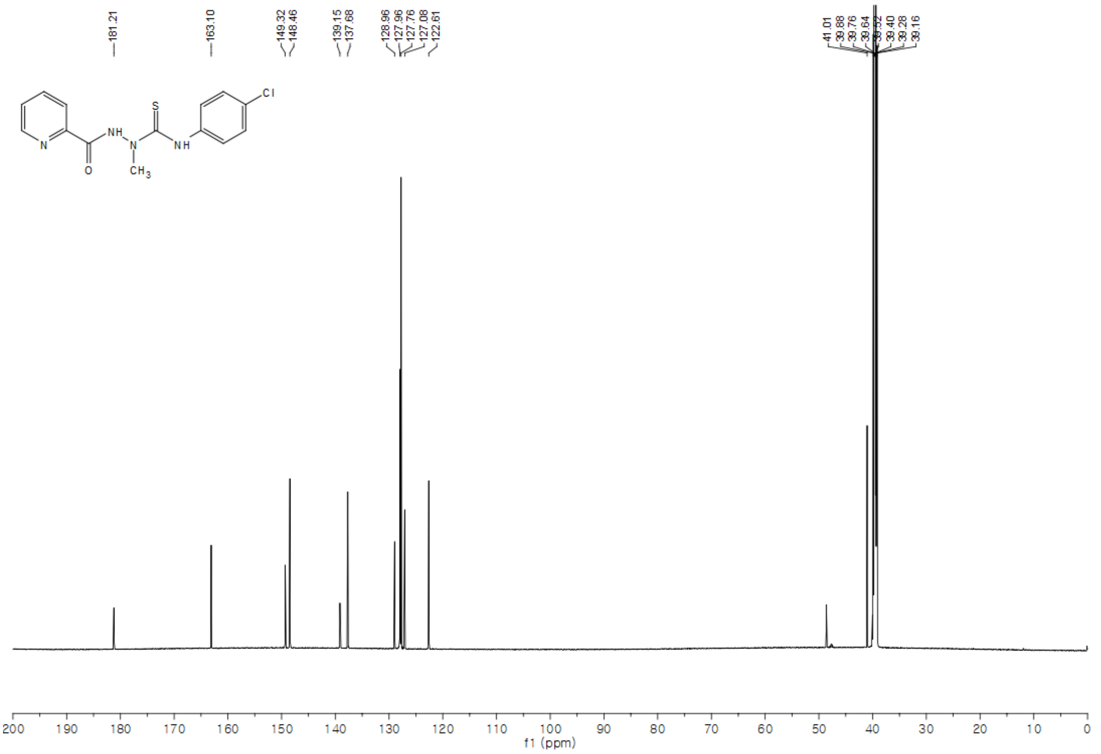

^{13}C-NMR spectrum of Compound **14**

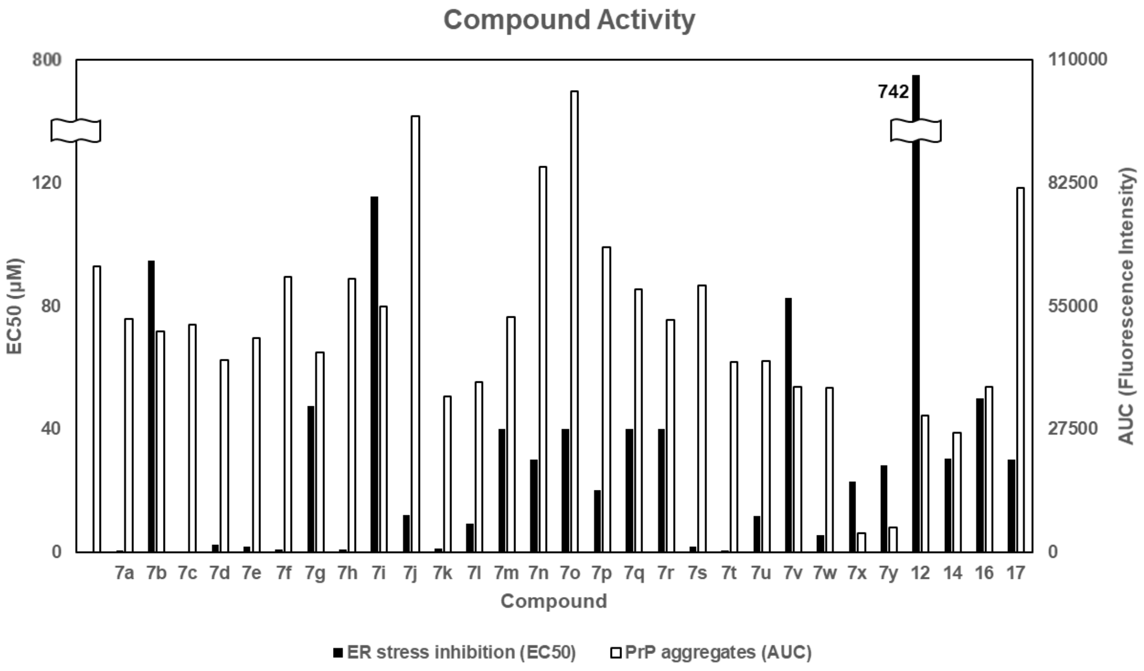

Supplementary figure 1. Comparison of ER stress inhibitory activity (EC_{50}) with anti-prion aggregation activity (AUC) of the compounds based on Figure 2F in text.

ER stress inhibition by compounds was measured by the method we have developed and reported elsewhere.[1,2] Briefly, synthesized human grp78 promoter (−137~+25) containing three ER stress response elements (ERSE) in tandem was cloned into pGL4.79 vector (Promega, Madison, USA). A stable human embryonic kidney (HEK 293) cell line harboring the reporter gene was selected under 400 μg/mL of G418 selection pressure. 5,000 cells/well were seeded in white 384-well plate (Greiner, Solingen, Germany). After 24 h, tunicamycin (Tm) or DMSO was added (1 μg/mL final concentration). Following 22 h incubation, 60 μM of EnduRen™ live cell substrate (Promega, Madison, USA) was added to medium and, after 2 h incubation, Renilla luciferase (rLuc) activity was measured using EnVision® (Perkin-Elmer). to normalize the rLuc activity, total viable cells were counted using Celltiter-glo™ reagent (Promega). The 50% inhibitory concentration (EC_{50}) values for tested compounds were determined by non-linear regression analysis of log-dose /response curves using

Prism® 5 software (GraphPad software Inc., CA, USA). Data from three independent experiments were expressed as the geometric mean EC_{50}.

References

1. Choi H, Yun W, Lee JH, et al. Synthesis and anti-endoplasmic reticulum stress activity of N-substituted-2-arylcarbonylhydrazinecabothioamides. Med Chem Res. 2019;28(12):2142–2152.

2. Kim YE, Kim DH, Choi A, et al. Bi-aryl analogues of salicylic acids: design, synthesis and SAR study to ameliorate endoplasmic reticulum stress. Drug Des Devel Ther. 2021;15:3593–3604.
